# Single-crystal quality data from polycrystalline samples: finding the needle in the haystack

**DOI:** 10.1107/S2052252523008163

**Published:** 2023-10-11

**Authors:** Joseph Charles Bear, Nikitas Terzoudis, Jeremy Karl Cockcroft

**Affiliations:** a School of Life Sciences, Pharmacy and Chemistry, Kingston University, Penrhyn Road, Kingston-upon-Thames, Surrey KT1 2EE, United Kingdom; b Department of Chemistry, University College London, 20 Gordon Street, London WC1H 0AJ, United Kingdom; Formby, Liverpool, United Kingdom

**Keywords:** multi-grain crystallography, co-crystals, heteroaromatic, *in situ* diffraction

## Abstract

Multi-grain crystallography, traditionally performed at synchrotron sources in association with high-pressure studies, has new relevance with respect to laboratory single-crystal X-ray diffraction, in which crystals can be grown rapidly *in situ*, and a preliminary dataset analysed and solved in a matter of minutes. Subsequently, a full-sphere of IUCr-quality data can then be collected in a few hours. To demonstrate the applicability of laboratory multi-grain crystallography with Cu *K*α X-rays, co-crystals of hexa­fluoro­benzene and pyrrole were grown rapidly by cooling a 1:1 liquid mixture in an X-ray capillary on the diffractometer. The software is able to identify a single unit cell from as few as 10% of the diffraction spots from a small number of diffraction frames. Once a unit cell is identified, a full crystal structure solution is rapidly obtained by collecting a small amount of data to a resolution of *ca* 1 Å. The co-crystal obtained from the 1:1 mixture showed that hexa­fluoro­benzene and pyrrole crystallize in a 3:4 ratio, in contrast to the columnar 1:1 adduct structures typified by hexa­fluoro­benzene and benzene. The generality of our multi-grain approach for samples that are liquid at room temperature (and form a polycrystalline solid mass on cooling) is further demonstrated by investigating and solving the 1:1 co-crystal formed between hexa­fluoro­benzene and pyridine.

## Abstract 2

Advances in X-ray hardware and software have revolutionized crystallography: now crystal structures can be routinely solved *first*, with publication-quality datasets collected *second*. This same approach now allows multi-grain crystallography to be completed in the laboratory with Cu *K*α X-rays.

## 1. Introduction

Single-crystal X-ray diffraction (SCXRD) is the gold-standard technique for structure determination in chemistry. The last decade has seen huge strides in hardware and software capability for SCXRD, with molecular structures being obtained routinely in minutes instead of hours or even days. On the hardware side, microfocus X-ray sources are now routinely available in the chemical laboratory with beam diameters focused down to about 100 µm compared with *ca* 0.5 mm a decade or so ago, allowing much smaller crystals to be studied. Solid-state detectors (hybrid-pixel photon counters), as developed by Dectris (Förster *et al.*, 2019 ▸) for example, have reduced data acquisition times dramatically due to their zero background (zero dark current) count compared with traditional charge-coupled device (CCD) detectors. As predicted by Moore’s Law, computer hardware used to process the data has changed equally dramatically over the last decade, *e.g.* an improvement in PC processors from an Intel 6 core I7 (3.3 GHz) in 2012 to an Intel 24 core I9 (5.8 GHz) in 2022; during this period, the most common operating system on PCs changed from Windows 7/8 to Windows 11.

Coupled with the changes in hardware, there have been synergistic strides forward in software. Data acquisition software such as *CrysAlis PRO* (Rigaku Oxford Diffraction, 2019 ▸), routinely performs concurrent unit-cell determination, data reduction to spot intensities *I*(*hkl*), and on-the-fly structure solution and refinement via automated software such as *AutoChem* within the graphical display tool *Olex2* (Dolomanov *et al.*, 2009 ▸; Matsumoto *et al.*, 2021 ▸). The latter depends on software for the structure solution of small chemical molecules, where the landscape has drastically changed from the popular use of programs such as *SHELXS* (Robinson & Sheldrick, 1988 ▸) and *Superflip* (Palatinus & Chapuis, 2007 ▸) to the dominant use of *SHELXT* (Sheldrick, 2015 ▸) today. The combination of these developments has led to significant changes in experimental strategy in our laboratory: the crystal structure is routinely solved *first*, using tools such as the ‘*What is this*?’ function (Matsumoto *et al.*, 2021 ▸) in *CrysAlis PRO*, and the publication-quality data is collected *second*. Although we used tools provided by Rigaku, we stress that the same approach is just as applicable using equipment and software from other X-ray manufacturers.

In recent years our laboratory has been investigating co-crystals comprising liquid co-formers at room temperature. This type of sample requires handling in X-ray capillaries due to the volatile nature of the components and cooled to the solid phase. Though it is possible in theory to grow a single-crystal at low temperature in the capillary, it is experimentally demanding to obtain just *one* crystal. This led us to the hypothesis: can advances in laboratory single-crystal hardware and software be used to determine crystal structures from a polycrystalline sample using low-energy (*i.e.* Cu *K*α) X-rays?

The concept of a multigrain approach is not in itself novel, at least with regard to data collected at the synchrotron on polycrystalline samples with high-energy X-rays. Although the concept of processing and solving structures from data resulting from the presence of two or more crystals goes back decades, as in the case of twinned crystals (*e.g.* Pratt *et al.*, 1971 ▸; Parsons, 2003 ▸), the development of ‘multigrain crystallography’ has been a more recent development, and now merits mention in the latest volume (H) of the International Tables for Crystallography (Gilmore *et al.*, 2019 ▸). Multigrain crystallography was developed for the analysis of bulk materials, such as metals and alloys in which individual grains may exhibit slightly different properties. When developed, it required the use of high-energy X-ray radiation for sample penetration and large-area detectors, both of which are typically available at X-ray synchrotron sources; indeed, most of the original studies were carried out using the high-energy X-ray beamline ID11 at the European Synchrotron Radiation Facility (ESRF) in Grenoble, France (Lauridsen *et al.*, 2001 ▸; Poulsen, 2004 ▸; Vaughan *et al.*, 2004 ▸; Sørensen *et al.*, 2012 ▸; Wejdemann & Poulsen, 2016 ▸). The concept of multigrain crystallography has proved particularly useful in the field of geological and material sciences, where there is a requirement to study phase transitions at high pressures using diamond anvil cells and synchrotron X-ray sources (Rosa *et al.*, 2015 ▸; Zhang *et al.*, 2019 ▸; Zurkowski *et al.*, 2022 ▸), and the need to characterize novel materials formed *in situ*, such as the formation of *cyclo*-N_5_^−^ pentazolate salts from sodium azide and N_2_ (Bykov *et al.*, 2021 ▸). Very recently, the software program *DAFi* was developed (and integrated into version 43 of *CrysAlis PRO*) to quickly find subsets of reflections from individual domains in a full sphere of SCXRD data measured under high-pressure conditions at synchrotron facilities (Aslandukov *et al.*, 2022 ▸). *DAFi* has also been tested by the authors with data from high-energy X-rays from a laboratory Ag source.

In this paper, we demonstrate the ease of solving and refining crystal structures when multiple crystals are present in the path of a Cu *K*α X-ray beam in the laboratory. This is important given the ubiquity of Cu laboratory sources. Moreover, the longer wavelength of Cu *K*α X-rays is advantageous compared with Mo or Ag sources as it increases the 2θ spacing of the diffraction spots, making unit-cell identification easier when multiple crystals are present in the beam.

We have decided to showcase our multi-grain approach in the context of our laboratory study into weak, non-covalent interactions in organic molecules. Weak, quadrupole–quadrupole interactions can be the dominant ordering force in the solid state in the absence of strong interactions, thus there is a need to understand and characterize weak interactions to aid crystal structure prediction. Interactions between aromatic rings, often dubbed ‘aromatic donor–acceptor’ interactions, are amongst the hardest to predict. Previous systems we have studied include 1:1 adducts of hexa­fluoro­benzene with benzene (Williams *et al.*, 1992 ▸; Cockcroft *et al.*, 2018 ▸), toluene, *p*-xylene (Cockcroft *et al.*, 2019 ▸), mesitylene (Cockcroft *et al.*, 2017 ▸) and more recently ferrocene (Bear *et al.*, 2020 ▸), all of which exhibit columnar structures with alternating aromatic molecules. More recently, we posed the question of what would happen to the structures of these adducts if we substituted one of the fluorides in C_6_F_6_ with hydrogen or another halide. In this paper, we discuss the adduct formed by C_6_F_5_H with *p*-xylene. Finally, we ask the question of whether potential columnar adducts might form from hexa­fluoro­benzene (C_6_F_6_) and simple heterocycles such as pyrrole (C_4_H_5_N) and pyridine (C_5_H_5_N).

## 2. Experimental

The first multigrain system described here is the solid formed at low temperature from C_6_F_6_ and C_4_H_5_N. We illustrate this one first as, somewhat unusually, the co-formers did not form a 1:1 co-crystal. Consequently, it proved to be more difficult to crystallize and obtain a structure solution with the components mixed in the wrong ratio. Therefore, it is an ideal example to illustrate the robust nature of our multigrain approach.

### 2.1. Sample preparation

Initially, we prepared a liquid sample comprising C_6_F_6_ and C_4_H_5_N in a 1:1 molar ratio as for a columnar adduct. An X-ray glass capillary (Ø = 0.4 mm, length 85 mm) was initially shortened by around 10–15 mm so as to fit into a Microhematocrit centrifuge. The neck of the capillary was charged with a drop of the liquid mixture via pipette and centrifuged for about 30 s at 360*g*. Great care must be taken not to smash or damage the capillary in the centrifuge before subsequent removal of the capillary neck by flame sealing. As noted previously, it is important for the capillary to be 100% sealed as even a micrometre-sized perforation in the capillary wall can result in slow loss of volatile components over time (Bear *et al.*, 2020 ▸). Further details of the materials used are given in the supporting information.

### 2.2. X-ray diffraction

After mounting on the SCXRD instrument, the capillary containing the C_6_F_6_:C_4_H_5_N was cooled rapidly *in situ* to 230 K resulting in the formation of numerous white crystals. The temperature was then raised until the crystals fully melted, before subsequent cooling back to 230 K. The temperature was then increased to 260 K, (just below the melting point at 266 K) in order to encourage annealing of single crystals present. The sample was held at 260 K for 2 h before cooling to 254 K for data acquisition. Initially, a full sphere of data was collected to *ca* 0.84 Å resolution using an Agilent Oxford Diffraction Supernova diffractometer upgraded with a Rigaku HyPix Arc-100 detector (Fig. S1 of the supporting information) in about 2.5 h. Subsequently, the sample was cooled to 150 K and a further full sphere of data was collected. Further details are given in the supporting information.

### 2.3. Differential scanning calorimetry

Differential scanning calorimetry (DSC) data were measured on a new sample of (C_6_F_6_)_3_:(C_4_H_5_N)_4_ prepared knowing the actual ratio of 3:4 for the components obtained from the SCXRD results. Further details are provided in the supporting information.

## 3. Results and discussion

### 3.1. Traditional practice versus many crystals in the beam

Single-crystal diffraction is exactly that: the concept of rotating just *one* crystal in an X-ray beam while measuring diffracted intensities, a technique which dates back to the earliest days of X-ray crystallography by Bragg & Bragg (1913 ▸). Traditionally, a single crystal is carefully selected with the aid of an optical microscope (ideally equipped with cross-polarizing filters) before mounting it onto a goniometer head prior to measurement. This requires a high degree of skill on the part of the crystallographer. Indeed, with the current generation of laboratory-based instruments setup with automatic data collection and reduction, and structure solution and refinement, the selection of the crystal is perhaps one of the few skills left!

But what does the crystallographer do when the sample is not a solid under ambient conditions? There are many chemical systems of interest where the substance under investigation is a liquid at room temperature, but exhibits one or more crystalline phases on cooling. The traditional approach is to try to grow a single crystal at low temperature, using a cooling/heating device with a very precise temperature controller. Examples of devices include the use of a focused halogen lamp, filtered for all but red light (Goddard *et al.*, 1997 ▸), or an infrared laser to melt a sample held in the capillary while holding the sample at a particular temperature (Thalladi *et al.*, 1998 ▸; Choudhury *et al.*, 2005 ▸). Although this approach has the advantage of potentially producing just one crystal in the X-ray beam, it has several drawbacks. The experimental setup with an infrared heating laser requires extra equipment inside the X-ray radiation enclosure, which involves additional expense and health and safety considerations. Secondly, the operation of a laser to grow a single crystal *in situ* requires a high degree of skill, with significant time expended on the part of the user. Alternatively, for some molecular systems, it may be possible if given enough time to grow a single-crystal *in situ* via repeated melting and cooling cycles (Yufit & Howard, 2010 ▸; Yufit *et al.*, 2012 ▸).

The difficulty of growing just one single crystal from a liquid in the laboratory can potentially be overcome by forming several crystals *in situ* and then analysing the X-ray diffraction data using the multigrain approach first developed at synchrotron facilities with hard X-ray sources. We found that the combination of using *CrysAlis PRO* for identifying a crystal of significant size and finding the correct unit cell, followed by *SHELXT* to rapidly solve the structure from a limited resolution dataset is a powerful tool in this regard.

**Figure 1:**
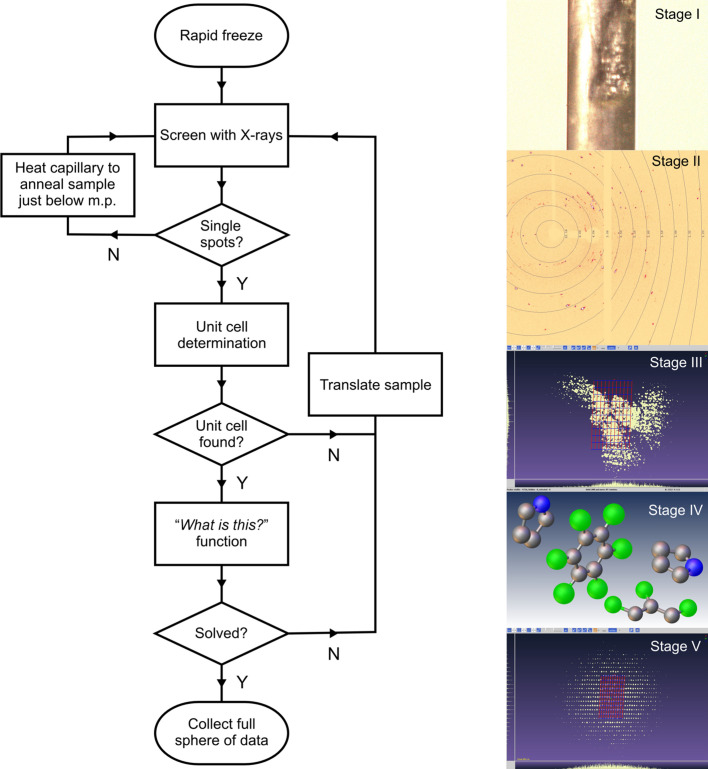
Flow diagram showing the overall process followed in our approach to obtaining single-crystal-quality data from a polycrystalline sample. The different stages of the process are illustrated on the right-hand side. Stage I illustrates the frozen capillary which is then screened with the X-ray beam (stage II) to test if large multiple crystals are present (as seen here) or whether the sample is simply a polycrystalline powder. If the unit-cell determination from the screening frames (stage III) is successful, data to 1.0 Å resolution is collected for an initial structure solution as shown in stage IV. The final stage is to obtain a full sphere of data to *ca* 0.84 Å resolution (stage V) for publication from which the final structure is refined.

Our approach with a liquid mixture, illustrated with a flow diagram in Fig. 1 ▸, is to cool the sample into the solid state and screen the capillary with X-rays (stages I and II). There are several possibilities: the sample forms large macroscopic crystals from the outset, it forms a large number of smaller crystallites, it forms a crystalline powder or it forms an amorphous solid. In our experience, these co-crystal systems rarely form ideal crystalline powders even when the sample is quenched at low temperature. This implies that the structures cannot be determined reliably from powder X-ray diffraction (PXRD) data. We note that, in our previous work on C_6_D_6_:C_6_F_6_, the sample was ground in liquid N_2_ so as to obtain an ideal powder and that simply taking a frozen sample of C_6_D_6_:C_6_F_6_ through three solid-state phase transitions does not achieve this, even with the large-volume samples typically used for powder neutron diffraction.

The key step in solving the crystal structure is to find the correct unit cell (Fig. 1 ▸, stage III). In this regard, success is more likely when the sample contains a small number of larger crystals rather than a large number of smaller ones. Initially, the sample is screened by collecting a few frames of diffraction data. Frames that exhibit fewer diffraction spots and diffraction spots to high angle (1 Å resolution or better) are indicative of a sample containing a small number of large crystals, whereas frames that exhibit a large number of spots (and which often lie in powder diffraction rings) are unlikely to yield sensible unit-cell solutions. Examples of data from usable and unusable frames are shown in Fig. S2.

If the sample is composed of only small crystals, several strategies are available. In order to encourage the growth of larger crystals, the sample can be warmed to just below the melt and annealed. This can promote the dissolution of smaller crystallites and growth of the larger ones. In addition, slow translation of the axis of the capillary through the cold gas of the Cryojet5 is facilitated by manual adjustment of the *z* axis of the goniometer head. Translation through this temperature gradient promotes crystal growth and the sample is re-screened.

Even with a sample containing large crystals, it is usually necessary to collect more screening frames than the typical 10 or 20 measured in a conventional single-crystal experiment in order to find potential unit cells. In our experience, software tools such as *CrysAlis PRO* can find chemically sensible unit cells from as little as *ca* 10% of the total number of diffraction peaks measured, but this percentage can be expected to reduce further in future with the introduction of the improved algorithms in programs such as *DAFi* (Aslandukov *et al.*, 2022 ▸). Peak-search software identifies the position of the diffraction peaks and these may be plotted in reciprocal space using tools such as the *Ewald Explorer* within *CrysAlis PRO* along with suggested unit cells (see Fig. S3). Unit cells attributed to genuine periodicity in reciprocal space can be identified from sharp peaks seen in the distribution histograms along the a*, b* and c* directions (see Fig. S4). In addition, a clear reciprocal lattice of spots should be observed when the unindexed (or ‘wrong’ spots) are removed from the display (see Figs. S4 and S5). Furthermore, only unit cells with reasonable unit-cell volumes consistent with combinations of the molecular volumes of the components need to be considered.

Finally, indexing solutions with unit cells with two or more cell angles of 90° (and with symmetry higher than triclinic) are often indicative of a cell that is crystallographically likely and which is improbable by random indexing of the spots. Finding the correct unit cell is the hardest step in the whole process since this is analogous to looking for the proverbial ‘needle in a haystack’ but not knowing *a priori* the shape of the needle. Obviously, once the structure has been solved, finding the correct unit cell again in a screening set of frames is relatively trivial.

Once a unit cell has been identified, a small amount of data can be collected to a resolution of *ca* 1 Å and the data processed to obtain the *F* ^2^(*hkl*) required for an initial structure solution using *SHELXT* (Fig. 1 ▸, stage IV). This whole process can be performed simply with built-in tools such as ‘*What is this?*’ in *CrysAlis PRO*, though we stress that it can be achieved easily by the crystallographer manually. Once a chemically sensible structure is obtained, the next step is to collect a full sphere of data to the minimum IUCr *d*-spacing resolution specification (Fig. 1 ▸, stage V). We choose to measure a full sphere to improve data reliability with regard to detrimental effects from absorption and accidental peak overlap. Although in principle software can process data from multiple crystals, in practice we found that processing the data from the one crystal that produces the most spots, and especially with spots at high angles, works best as this will correspond to the largest crystal in the multigrain sample. In principle, data from smaller crystals in the beam may be analysed and the data merged, but this may not improve the overall quality of the data significantly. In our approach, data from the smaller crystals is simply binned. In addition, when using a capillary that is wider than the X-ray beam, a crystal that produces a full sphere of spots is likely to correspond to one that is fully bathed by the beam. The final step is to refine the crystal structure from conventional single-crystal diffraction data.

### 3.2. The scientific problem

In order to test our hypothesis concerning the applicability of a multigrain approach with laboratory Cu *K*α X-ray diffraction data, we investigated co-crystals of C_6_F_6_ with aromatic heterocyclic co-formers. We have been investigating such systems to probe weak, non-covalent interactions. This is achieved by combining neutral organic molecules with positive (C_6_F_6_) and negative [*e.g.* benzene C_6_H_6_, *p*-xylene C_6_H_4_Me_2_, ferrocene FeCp_2_ where (Cp = C_5_H_5_)] molecular quadrupolar moments, so as to maximize the electrostatic interaction between faces of the aromatic rings and thus generate a face-to-face pairing of the planar molecules, which can lead to closely packed columns of alternating molecules. Our previous report into the isolation and discovery of the C_6_F_6_:FeCp_2_ co-crystal demonstrated the viability of the columnar stacking of five and six-membered aromatic systems into binary adducts (Bear *et al.*, 2020 ▸).

Therefore, we sought to determine whether five-membered aromatic heterocycles would exhibit similar columnar stacking behaviour when used as a co-former with C_6_F_6_. *Prima facie* evidence suggested that pyrrole (C_4_H_5_N) would be an ideal candidate. Pyrrole is an air-stable, five-membered aromatic heterocycle with a negative molecular quadrupole moment, whose magnitude is expected to be comparable to the positive molecular quadrupole moment of C_6_F_6_ (+32 × 10^−40^ C m^2^) determined by Battaglia *et al.* (1981 ▸). In our previous studies, the co-formers chosen have a dipole moment equal to either zero or a small positive value, *e.g.* toluene with a moment equal to 0.33 D (Cumper *et al.*, 1969 ▸). By contrast, owing to the presence of the highly electronegative nitro­gen heteroatom in the aromatic ring, pyrrole possesses a large dipole moment of 1.74 D (Bohn *et al.*, 1989 ▸).

In our SCXRD experiments, a 1:1 molar ratio of C_6_F_6_:C_4_H_5_N (as a liquid mixture) was added into a Ø = 0.4 mm capillary and cooled *in situ* with the Cryojet5 on the X-ray diffractometer to 230 K; this temperature was chosen as it is below the freezing points of the pure components. Moreover, the SCXRD is equipped with a camera, so real-time observation of freezing is possible (Fig. 1 ▸, stage I). The sample was then melted and re-frozen, before being increased to 260 K (just below the melting point at 266 K) to allow a degree of ‘annealing’ in order to grow larger (single) crystallites. It is noteworthy that in this study both co-formers are liquid at room temperature. If there is a significant difference in melting point of the two co-formers, then it may be possible to dissolve one co-former into the other, *e.g.* as in the formation of C_6_F_6_:Fe(C_5_H_5_)_2_ from Fe(C_5_H_5_)_2_ dissolved in an excess of C_6_F_6_ (Bear *et al.*, 2020 ▸). However, this approach works well when the adduct can be isolated, but becomes problematic when the excess of the co-former solvent is frozen at low temperature leading to additional X-ray diffraction scattering.

**Figure 2:**
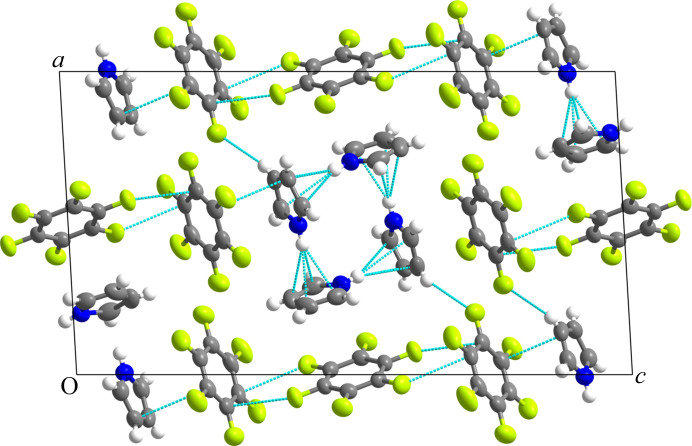
Refined crystal structure of (C_6_F_6_)_3_:(C_4_H_5_N)_4_ in space group *P*2_1_/*n* at 150 K viewed down *b*. Thermal ellipsoids are shown at 50% probability using the program *Mercury* from the CCDC (Macrae *et al.*, 2008 ▸). The dashed lines in cyan show the closest contacts as identified by *Mercury*. The crystallographic labelling of the atoms is shown in Fig. S6.

Screening of the sample revealed multiple single crystals (Fig. 1 ▸, stage II); however, the software was able to identify a monoclinic unit cell with a volume equal to 1795 Å^3^ based on as few as 12.5% of the spots, and a cell which is unlikely to occur by random indexing of the spots. However, the relatively large volume of 1795 Å^3^, which suggested approximately seven molecules per unit cell (assuming a volume of around 160 Å^3^ for C_6_F_6_ and 90 Å^3^ for C_4_H_5_N), was less encouraging. Nevertheless, the ‘*What is this?*’ function was used to find an approximate solution for the structure using this cell (Fig. 1 ▸, stage III). Surprisingly, it was found that the structure crystallized in a 3:4 molar ratio of C_6_F_6_:C_4_H_5_N *despite* the 1:1 molar ratio mixture from which the crystals were grown (Fig. 1 ▸, stage IV). Thus, the result is consistent with the number of molecules per unit cell determined from the cell volume of 1795 Å^3^. A full sphere of data was then collected to 0.84 Å at 254 K and subsequently at 150 K (Fig. 1 ▸, stage V). Details of the data processing and crystal structure refinement are given in the supporting information, with full crystallographic details in Tables S1(*a*)–S1(*e*) and in the deposited CIF. The crystal structure of (C_6_F_6_)_3_:(C_4_H_5_N)_4_ at 150 K is shown in Fig. 2 ▸.

Once the full sphere of data was collected at 150 K, the peak search and auto unit-cell determination identified a similar unit cell to that found at 254 K with about 13% of the total diffraction spots attributable to just one crystal. Data reduction resulted in processing a value of *R*_int_ equal to about 10%. With the multigrain approach, a degree of random overlap of diffraction spots from other crystals is expected and this may lead to higher than normal values of *R*_int_. In this instance, where the crystals of (C_6_F_6_)_3_:(C_4_H_5_N)_4_ were grown from a solution containing the components in an incorrect ratio, so that excess C_6_F_6_ is present, there is a greater degree of overlap of diffraction spots from solid C_6_F_6_. However, the relatively high value of *R*_int_ obtained does not result in a poor crystal structure and, consequently, we considered that there was no need to repeat the experiment with a sample containing the constituents in the correct 3:4 ratio.

The high quality of the structure determination is shown by the values of the bond lengths and angles obtained for the C_6_F_6_ and C_4_H_5_N rings [Tables S1(*d*) and S1(*e*)]: the average values of the C—C and C—F bonds in C_6_F_6_, namely 1.372 (5) and 1.337 (5) Å, respectively, are comparable to those observed in pure C_6_F_6_ at 132 K, namely 1.382 (2) and 1.332 (2) Å (Rusek *et al.*, 2020 ▸). The flatness of the aromatic hexagonal ring is demonstrated by the average internal (C—C—C) bond angle of 120.0 (4)°, which is equal to the ideal value of 120°, average torsion angles very close to 0 or 180°, and mean plane deviations for the two rings in the asymmetric unit equal to 0.002 and 0.008°. By comparing the C—C and C—N bond lengths in the C_4_H_5_N ring with those determined previously for pure C_4_H_5_N at 103 K (Goddard *et al.*, 1997 ▸), we found the average deviation is only 0.022 Å; furthermore, the average sum of the internal angles within the pentagonal ring of 540° is a perfect match for that expected for a flat pentagonal ring, as is the average ring torsion angle of 0.4 (2)° with mean plane deviations (excluding hydrogen atoms) for the two rings in the asymmetric unit equal to 0.002 and 0.003°. In addition, the quality of the data is such that all ten hydrogen atoms for the two crystallographically distinct pyrrole rings in the unit cell are observed as the ten highest peaks in the difference Fourier map (as shown in Fig. S7) and all ten hydrogen atoms can be refined as independent isotropic atoms without the need for constraints.

The crystal structure formed by C_6_F_6_ and C_4_H_5_N is not of the adduct type as observed in crystal structures like C_6_F_6_:C_6_H_6_ (Cockcroft *et al.*, 2018 ▸) but shows a mixture of face and edge interactions between the aromatic rings. It is interesting to compare the interactions between pyrrole rings in this co-crystal with those observed in the solid structure of pyrrole (Goddard *et al.*, 1997 ▸). In pyrrole, the molecules interact to form zigzag chains linked by an ‘N—H⋯π hydrogen bonding’ interaction whereas in (C_6_F_6_)_3_:(C_4_H_5_N)_4_ the pyrrole molecules assemble into distinctive tetramer units in which the hydrogen on each nitrogen atom is directed more towards the centre of the neighbouring pyrrole ring (see Fig. S8). This raises the interesting question of whether such units form in the liquid phase. In these tetramer units, the molecular dipoles are opposed such that the net dipole is zero. In addition to the pyrrole self-interactions, there is also an orthogonal interaction between the edges of C_6_F_6_ rings. Note that in pure C_4_H_5_N and in (C_6_F_6_)_3_:(C_4_H_5_N)_4_ there is no possibility for a ‘donor N—H⋯acceptor’ type interaction. Electron density and Hirshfeld 3D surface maps were calculated with the program *CrystalExplorer* (Spackman *et al.*, 2021 ▸) (Fig. S9). The electron density calculated was close to what might be anticipated, showing the expected deviations from the pure compounds as a result of the intermolecular interactions between them that form the co-crystal. The Hirshfeld surface is particularly useful in illustrating the closest contacts between the rings.

DSC data on a freshly prepared sample of (C_6_F_6_)_3_:(C_4_H_5_N)_4_ with components in the correct 3:4 ratio demonstrated the absence of phase transitions as a function of temperature (Fig. S10) in contrast to a number of similar systems we have studied (Cockcroft *et al.*, 2017 ▸, 2018 ▸, 2019 ▸). When phase transitions are present, the multigrain approach as used here may still be useful, *e.g.* when one or more crystals breaks into several smaller pieces, though in other instances the diffraction data resulting from it may exhibit broader peaks due to mosaic broadening from stress and/or strain effects. In some cases, twinning of the crystals on going through a low-temperature phase transition may present an additional problem. Under such circumstances, it may not be possible to obtain reliable peak intensities *I*(*hkl*) from the data frames as the diffraction spots become smeared out around the path of Debye–Scherrer diffraction rings.

### 3.3. Wider application

The successful structure determination of the co-crystal (C_6_F_6_)_3_:(C_4_H_5_N)_4_ posed several questions: would a mixture of C_6_F_6_ with other nitro­gen-containing monoheterocyclic aromatic rings form a co-crystal, and could we demonstrate that our laboratory multigrain approach could be used more widely, for example, with co-formers that are likely to form a columnar adduct which typically exhibit acicular crystal forms?

#### 3.3.1. C_6_F_6_:C_5_H_5_N

**Figure 3:**
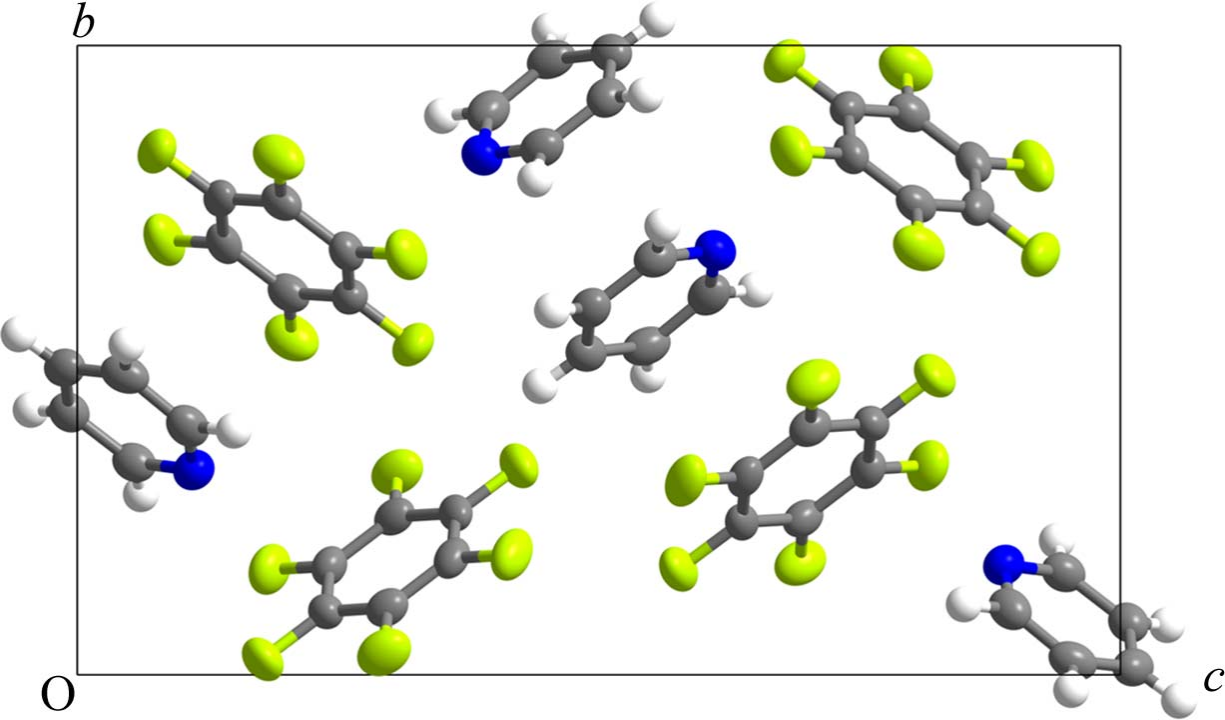
Refined crystal structure of C_6_F_6_:C_5_H_5_N in the space group *P*2_1_2_1_2_1_ at 150 K viewed down *a*. Thermal ellipsoids are shown at 50% probability using the program *Mercury* from the CCDC (Macrae *et al.*, 2008 ▸). Crystallographic labelling of the atoms is provided in Fig. S12.

Pyridine (C_5_H_5_N) is a liquid at room temperature and, like pyrrole, possesses a large dipole moment (2.12 D; Linde, 2004 ▸) with a negative quadrupole moment, again whose magnitude is expected to be comparable to the positive quadrupole moment of C_6_F_6_ (+32 × 10^−40^ C m^2^). As for our initial preparation of a sample of pyrrole:hexa­fluoro­benzene, a 1:1 molar mixture was prepared and inserted into an X-ray capillary. In light of previous experiments, one experimental improvement considered was the use of a narrower 0.2 mm capillary as shown in Fig. S11 so that there is less risk of the X-ray beam hitting a large crystal on the inner wall of the capillary, which may not be fully bathed by the beam in all orientations. Previously, we had used wider capillaries as they are easier to fill. However, a commercial capillary centrifuge enables liquid to be forced more easily to the end of a narrow capillary. This approach reduces the amount of sample in the path of the X-ray beam and potentially helps unit-cell identification by reducing the number of diffracting crystals in the beam. Details of the experimental procedure, data processing and crystal structure refinement are given in the supporting information, with full crystallographic details in Tables S2(*a*)–S2(*e*) and in the deposited CIF. The crystal structure of C_6_F_6_:C_5_H_5_N at 150 K is shown in Fig. 3 ▸.

This material proved more tractable regarding structure solution from a multigrain sample, in part due to a smaller capillary, but also due to the fact that the solid proved to be a 1:1 co-crystal. After screening the crystal, the software suggested a higher symmetry cell than for (C_6_F_6_)_3_:(C_4_H_5_N)_4_ and with a volume of just 1090 Å^3^, consistent with four units of C_6_F_6_:C_5_H_5_N in a primitive orthorhombic cell. A full sphere of data was then collected to *ca* 0.84 Å at 200 K and subsequently at 150 K. For the data at 150 K, the value of *R*_int_ (equal to about 6%) was significantly better than that obtained for (C_6_F_6_)_3_:(C_4_H_5_N)_4_. It is noteworthy that the quality of the refined crystal structure of C_6_F_6_:C_5_H_5_N is broadly similar to that of (C_6_F_6_)_3_:(C_4_H_5_N)_4_, with mean plane deviations for the C_6_F_6_ and C_5_H_5_N rings equal to 0.006 and 0.002°, respectively.

The structure formed by C_6_F_6_ and C_5_H_5_N is a 1:1 co-crystal, but is also not columnar in nature. Although the structure is not centrosymmetric, the orthorhombic space group symmetry of *P*2_1_2_1_2_1_ results in a net dipole moment of zero for the pyridine molecules. Further, there are no particularly close contacts and there is no possibility for an N—H donor⋯acceptor-type interaction due to the absence of a donor hydrogen on the heteroatom. As for the co-crystal formed by hexa­fluoro­benzene and pyrrole, electron density and Hirshfeld 3D surface maps were calculated for hexa­fluoro­benzene and pyridine and showed nothing unexpected (Fig. S13).

#### 3.3.2. *p*-C_6_H_4_Me_2_:C_6_F_5_H

**Figure 4:**
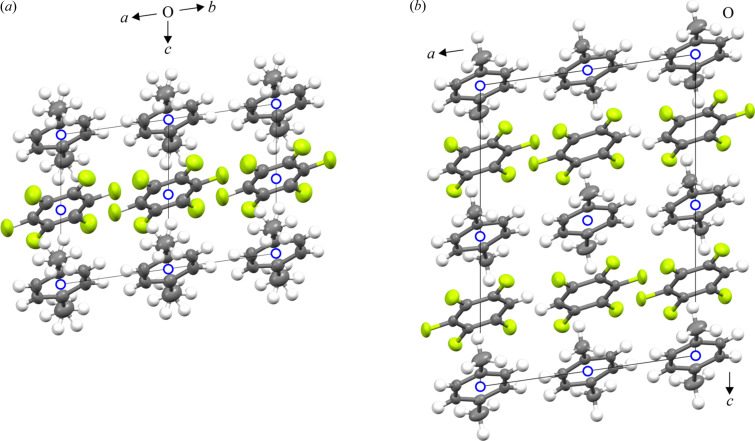
Comparison of the refined triclinic crystal structures of *p*-C_6_H_4_Me_2_:C_6_F_5_H at 160 K (phase I, left) and 120 K (phase II, right) with the molecular column axis set along *c*. Thermal ellipsoids are shown at 50% probability (except for hydrogen atoms which have a fixed radius) using the program *Mercury* from the CCDC (Macrae *et al.*, 2008 ▸). Both structures are triclinic and centrosymmetric, but on cooling, the C_6_F_5_H molecules order leading to a doubling of the structure along the column axis (*i.e.* the **c** direction) and loss of the crystallographic centre of symmetry (shown as blue open circles) at the middle of the C_6_F_5_H ring. To enable an easier comparison between the two structures as shown here, phase II was refined in space group *I* 1 with a unit cell 4× the volume of phase I. Crystallographic labelling of the atoms is given in Figs. S17 and S18.

To further demonstrate the wider applicability of the method outlined herein, we chose to analyse a potential columnar adduct system between two co-formers, namely penta­fluoro­benzene (C_6_F_5_H) and *p*-xylene, where the crystal form is expected to be non-block-like in contrast to the previous examples. We previously studied *p*-C_6_H_4_Me_2_:C_6_F_6_ which exhibits two order–disorder solid-state phase transitions at low temperature (Cockcroft *et al.*, 2019 ▸). Here we demonstrate that our method may still work even when the crystals have been taken through a phase transition and when the crystal system is triclinic. Identifying the correct unit cell under these conditions is more challenging but is still possible. Variable-temperature PXRD data on *p*-C_6_H_4_Me_2_:C_6_F_5_H clearly show a phase transition (Figs. S14 and S15), which is also shown in DSC measurements (Fig. S16). Phase I exists between 127 K and the melt at 253 K, with phase II existing below 127 K. Details of the PXRD and DSC experiments are given in the supporting information, along with the SCXRD experimental procedures, data processing and crystal structure refinement. It is noteworthy that the method worked well despite the fact that the data were collected with our older CCD detector prior to the upgrade to the hybrid-pixel photon detector. Full crystallographic details are given in Tables S3(*a*)–S3(*e*) and S4(*a*)–S4(*e*) and in the deposited CIFs. The crystal structures of the two phases of *p*-C_6_H_4_Me_2_:C_6_F_5_H are shown in Fig. 4 ▸.

Fig. 4 ▸ shows disordered C_6_F_5_H molecules in phase I (in which the hydrogen has six possible positions) and ordered molecules in phase II (in which the hydrogen has just one position), the latter resulting in a doubling of the unit cell along the column axis direction **c**. The structures shown in Fig. 4 ▸ are somewhat idealized: at 120 K the C_6_F_5_H still has some residual disorder and at 160 K the position of the lone hydrogen atom is not distributed equally on each of the fluorine atom positions (and so cannot be seen in the figure). Nonetheless, the quality of the refined crystal structures of *p*-C_6_H_4_Me_2_:C_6_F_5_H in both phases is high and is broadly similar to that of the two previous examples. This is demonstrated by ring flatness, where torsion angles are close to their ideal values, and mean plane deviations which, as determined by *Olex2*, vary from 0.000 to 0.003 Å in *p*-C_6_H_4_Me_2_ and from 0.008 to 0.012 Å in C_6_F_5_H. For the *p*-C_6_H_4_Me_2_ molecule, the methyl groups are shown to be disordered in phase I and ordered in phase II.

With regard to the phase II to phase I transition in *p*-C_6_H_4_Me_2_:C_6_F_5_H, we note that DSC provides an enthalpy of transition of about 0.3 kJ mol^−1^. This value is of similar magnitude to the low-temperature order–disorder phase transitions observed in C_6_H_5_Me:C_6_F_6_ and *p*-C_6_H_4_Me_2_:C_6_F_6_, both of which result from changes in orientational order–disorder of the methyl group in these columnar adducts (Cockcroft *et al.*, 2019 ▸). This raises the question of whether the ordering of the C_6_F_5_H molecules along the column axis is coupled to the ordering of the methyl groups in *p*-C_6_H_4_Me_2_. Since only a *single* solid-state transition was observed by DSC at low temperature, we postulate that this is indeed the case.

## 4. Conclusions

In answer to our hypothesis ‘can the advances in laboratory single-crystal hardware and software be used to determine crystal structures from a polycrystalline sample using low-energy (*i.e.* Cu *K*α) X-rays?’ then the answer is clearly yes. The multigrain approach highlighted here is a rapid and facile approach that has the capability to determine solid-state structures of materials that are volatile or liquid at room temperature. Moreover, it may be possible to determine the structure when the material has passed through a solid-state phase transition that results in multiple crystals in the sample when single crystals fracture. However, the method does have its drawbacks. The overall quality of the data will naturally not be as good as that obtained from a perfect single crystal. However, very few reflections required omission from the least-squares refinement in the examples provided here. With multiple crystals in the beam, a large number of overlapping spots will potentially lead to higher values of *R*_int_. However, this is ameliorated to some extent by collection of a full sphere of data with many different X-ray paths through the sample. Potentially, higher values of *R*_int_ can introduce larger standard deviations into the parameters of the refined structure, but in our experience, the slightly higher uncertainties have no significant detrimental effect as demonstrated by the values of the molecular parameters we obtained. The method has a lot of potential for the study of molecular systems that are liquid at room temperature: we have solved and refined the structures of over a dozen other similar co-crystals using this method, some of which exhibit phase transitions, and all form the subject of other papers now in preparation. Finally, we anticipate that the method is unlikely to work well for materials that crystallize in high-symmetry space groups, but as the majority of organic and inorganic materials crystallize in triclinic, monoclinic or orthorhombic crystal systems, we do not see this as a major limitation.

## Supplementary Material

Crystal structure: contains datablock(s) exp_197, exp_299, exp_3163, exp_3166. DOI: 10.1107/S2052252523008163/lt5060sup1.cif

Structure factors: contains datablock(s) exp_197. DOI: 10.1107/S2052252523008163/lt5060exp_197sup2.hkl

Structure factors: contains datablock(s) exp_299. DOI: 10.1107/S2052252523008163/lt5060exp_299sup3.hkl

Structure factors: contains datablock(s) exp_3163. DOI: 10.1107/S2052252523008163/lt5060exp_3163sup4.hkl

Structure factors: contains datablock(s) exp_3166. DOI: 10.1107/S2052252523008163/lt5060exp_3166sup5.hkl

Supporting data, figures and tables. DOI: 10.1107/S2052252523008163/lt5060sup6.pdf

CCDC references: 2263896, 2263897, 2287266, 2287267

## References

[bb1] Aslandukov, A., Aslandukov, M., Dubrovinskaia, N. & Dubrovinsky, L. (2022). *J. Appl. Cryst.* **55**, 1383–1391. 10.1107/S1600576722008081 PMC9533752 36249501

[bb2] Battaglia, M. R., Buckingham, A. D. & Williams, J. H. (1981). *Chem. Phys. Lett.* **78**, 421–423.

[bb3] Bear, J. C., Cockcroft, J. K. & Williams, J. H. (2020). *J. Am. Chem. Soc.* **142**, 1731–1734. 10.1021/jacs.9b11895 31927990

[bb4] Bohn, R. K., Hillig, K. W. & Kuczkowski, R. L. (1989). *J. Phys. Chem.* **93**, 3456–3459.

[bb5] Bragg, W. H. & Bragg, W. L. (1913). *Proc. R. Soc. London Ser. A*, **88**, 428–438.

[bb6] Bykov, M., Bykova, E., Chariton, S., Prakapenka, V. B., Batyrev, I. G., Mahmood, M. F. & Goncharov, A. F. (2021). *Dalton Trans.* **50**, 7229–7237. 10.1039/d1dt00722j 33913993

[bb7] Choudhury, A. R., Winterton, N., Steiner, A., Cooper, A. I. & Johnson, K. A. (2005). *J. Am. Chem. Soc.* **127**, 16792–16793. 10.1021/ja055956u 16316218

[bb8] Cockcroft, J. K., Ghosh, R. E., Shephard, J. J., Singh, A. & Williams, J. H. (2017). *CrystEngComm*, **19**, 1019–1023.

[bb9] Cockcroft, J. K., Li, J. G. Y. & Williams, J. H. (2019). *CrystEngComm*, **21**, 5578–5585.

[bb10] Cockcroft, J. K., Rosu-Finsen, A., Fitch, A. N. & Williams, J. H. (2018). *CrystEngComm*, **20**, 6677–6682.

[bb11] Cumper, C. W. N., Melnikoff, A. & Rossiter, R. F. (1969). *Trans. Faraday Soc.* **65**, 2892–2899.

[bb12] Dolomanov, O. V., Bourhis, L. J., Gildea, R. J., Howard, J. A. K. & Puschmann, H. (2009). *J. Appl. Cryst.* **42**, 339–341.

[bb13] Förster, A., Brandstetter, S. & Schulze-Briese, C. (2019). *Phil. Trans. R. Soc. A.* **377**, 20180241. 10.1098/rsta.2018.0241 PMC6501887 31030653

[bb16] Gilmore, C. J., Kaduk, J. A. & Schenk, H. (2019). Editors. International Tables for Crystallography: Powder Diffraction, Vol. H, 1st ed., pp. 606–607 Chester: International Union of Crystallography.

[bb14] Goddard, R., Heinemann, O. & Krüger, C. (1997). *Acta Cryst.* C**53**, 1846–1850.

[bb17] Lauridsen, E. M., Schmidt, S., Suter, R. M. & Poulsen, H. F. (2001). *J. Appl. Cryst.* **34**, 744–750.

[bb15] Linde, D. R. (2004). Editor. *Handbook of Chemistry and Physics*, 85th ed. pp. 15–24. Danvers, MA: CRC Press.

[bb18] Macrae, C. F., Bruno, I. J., Chisholm, J. A., Edgington, P. R., McCabe, P., Pidcock, E., Rodriguez-Monge, L., Taylor, R., van de Streek, J. & Wood, P. A. (2008). *J. Appl. Cryst.* **41**, 466–470.

[bb19] Matsumoto, T., Yamano, A., Sato, T., Ferrara, J. D., White, F. J. & Meyer, M. (2021). *J. Chem. Crystallogr.* **51**, 438–450.

[bb20] Palatinus, L. & Chapuis, G. (2007). *J. Appl. Cryst.* **40**, 786–790.

[bb21] Parsons, S. (2003). *Acta Cryst.* D**59**, 1995–2003. 10.1107/s0907444903017657 14573955

[bb22] Poulsen, H. F. (2004). *Multigrain Crystallography* in *Three-Dimensional X-ray Diffraction Microscopy.* Springer Tracts in Modern Physics, **Vol. 205**, pp. 83–88.

[bb23] Pratt, C. S., Coyle, B. A. & Ibers, J. A. (1971). *J. Chem. Soc. A*, pp. 2146.

[bb101] Rigaku Oxford Diffraction (2019). *CrysAlis PRO*. Rigaku Oxford Diffraction, Neu-Isenburg, Germany.

[bb24] Robinson, W. T. & Sheldrick, G. M. (1988). *Crystallographic Computing 4: Techniques and New Technologies*, edited by Isaacs, N. W. & Taylor, M. R. pp. 366–377. IUCr/Oxford University Press.

[bb25] Rosa, A. D., Hilairet, N., Ghosh, S., Garbarino, G., Jacobs, J., Perrillat, J.-P., Vaughan, G. & Merkel, S. (2015). *J. Appl. Cryst.* **48**, 1346–1354.

[bb26] Rusek, M., Kwaśna, K., Budzianowski, A. & Katrusiak, A. (2020). *J. Phys. Chem. C*, **124**, 99–106.

[bb27] Sheldrick, G. M. (2015). *Acta Cryst.* A**71**, 3–8.

[bb28] Sørensen, H. O., Schmidt, S., Wright, J. P., Vaughan, G. B. M., Techert, S., Garman, E. F., Oddershede, J., Davaasambuu, J., Paithankar, K. S., Gundlach, C. & Poulsen, H. F. (2012). *Z. Kristallogr.* **227**, 63–78.

[bb29] Spackman, P. R., Turner, M. J., McKinnon, J. J., Wolff, S. K., Grimwood, D. J., Jayatilaka, D. & Spackman, M. A. (2021). *J. Appl. Cryst.* **54**, 1006–1011. 10.1107/S1600576721002910 PMC8202033 34188619

[bb30] Thalladi, V. R., Weiss, H.-C., Bläser, D., Boese, R., Nangia, A. & Desiraju, G. R. (1998). *J. Am. Chem. Soc.* **120**, 8702–8710.

[bb31] Vaughan, G. B. M., Schmidt, S. & Poulsen, H. F. (2004). *Z. Kristallogr.* **219**, 813–825.

[bb32] Wejdemann, C. & Poulsen, H. F. (2016). *J. Appl. Cryst.* **49**, 616–621. 10.1107/S1600576716003691 PMC4815876 27047308

[bb33] Williams, J. H., Cockcroft, J. K. & Fitch, A. N. (1992). *Angew. Chem. Int. Ed. Engl.* **31**, 1655–1657.

[bb34] Yufit, D. S. & Howard, J. A. K. (2010). *CrystEngComm*, **12**, 737–741.

[bb35] Yufit, D. S., Zubatyuk, R., Shishkin, O. V. & Howard, J. A. K. (2012). *CrystEngComm*, **14**, 8222–8227.

[bb36] Zhang, L., Yuan, H., Meng, Y. & Mao, H.-K. (2019). *Engineering*, **5**, 441–447.

[bb37] Zurkowski, C. C., Lavina, B., Brauser, N. M., Davis, A. H., Chariton, S., Tkachev, S., Greenberg, E., Prakapenka, V. B. & Campbell, A. J. (2022). *Am. Mineral.* **107**, 1878–1885.

