# Supplementary material for: Single-crystal quality data from polycrystalline samples: finding the needle in the haystack
Source: IUCrJ. 2023 Oct 11;10(Pt 6):720–8. doi: 10.1107/S2052252523008163 (PMC10619455; doi:10.1107/S2052252523008163)
Supplement: Supplementary file 6 [file m-10-00720-sup6.pdf]

IUCrJ

Volume 10 (2023)

Supporting information for article:

Single-crystal quality data from polycrystalline samples: finding the needle in the haystack

Joseph Charles Bear, Nikitas Terzoudis and Jeremy Karl Cockcroft

Single−crystal quality data from polycrystalline samples: finding the needle in the haystack

Joseph Charles Bear[a], Nikitas Terzoudis[a] and Jeremy Karl Cockcroft[b]*

[a]School of Life Sciences, Pharmacy and Chemistry, Kingston University, Penrhyn Road, Kingston−upon−Thames, Surrey, KT1 2EE, United Kingdom

[b]Department of Chemistry, University College London, 20 Gordon Street, London, WC1H 0AJ, United Kingdom

Correspondence email: j.k.cockcroft@ucl.ac.uk

Supporting information

Table of Contents | Page No.

Additional Experimental Details

S1. Sample preparation S3

S2. Single-crystal X-ray diffraction (SXD) experiments S3

S3. Powder X-ray diffraction (PXRD) experiments S6

S4. Differential scanning calorimetry (DSC) S6

S5. Hirshfeld surface calculations S7

List of Tables

SXD data for $(C_6F_6)_3$:$(C_4H_5N)_4$ at 150 K

Table S1a. Crystal data and structure refinement S8

Table S1b. Fractional atomic coordinates and U(eq) for all atoms S9

Table S1c. Anisotropic displacement parameters S10

Table S1d. Selected bond lengths S11

Table S1e. Selected bond angles S11

SXD data for C_6F_6:C_5H_5N at 150 K

Table S2a. Crystal data and structure refinement S12

Table S2b. Fractional atomic coordinates and U(eq) for all atoms S13

Table S2c. Anisotropic displacement parameters S14

Table S2d. Selected bond lengths S14

Table S2e. Selected bond angles S14

SXD data for phase I of *p*-$C_6H_4Me_2$:C_6F_5H at 160 K

Table S3a. Crystal data and structure refinement S15

Table S3b. Fractional atomic coordinates and U(eq) for all atoms S16

Table S3c. Anisotropic displacement parameters S17

Table S3d. Selected bond lengths S17

Table S3e. Selected bond angles S17

SXD data for phase II of *p*-$C_6H_4Me_2$:C_6F_5H at 120 K

Table S4a. Crystal data and structure refinement S18

Table S4b. Fractional atomic coordinates and U(eq) for all atoms S19

Table S4c. Anisotropic displacement parameters S20

Table S4d. Selected bond lengths S20

Table S4e. Selected bond angles S21

VT-PXRD data on *p*-$C_6H_4Me_2$:C_6F_5H

Table S5. Unit cell parameters as a function of temperature S22

List of Figures

Fig. S1. Photograph of the diffractometer S23

Fig. S2. Good versus bad raw X-ray data for $(C_6F_6)_3$:$(C_4H_5N)_4$ S24

Fig. S3. Ewald explorer figure of peaks in reciprocal space S25

Fig. S4. Indexed peaks in reciprocal space S26

Fig. S5. Whole sphere of data in reciprocal space S26

Fig. S6. Labelled atoms in the crystal structure of $(C_6F_6)_3$:$(C_4H_5N)_4$ S27

Fig. S7. Peaks from difference Fourier map for $(C_6F_6)_3$:$(C_4H_5N)_4$ S28

Fig. S8. Interactions in pure pyrrole versus the co-crystal $(C_6F_6)_3$:$(C_4H_5N)_4$ S29

Fig. S9. Electron density and Hirshfeld surface for $(C_6F_6)_3$:$(C_4H_5N)_4$ S30

Fig. S10. DSC data on $(C_6F_6)_3$:$(C_4H_5N)_4$ S31

Fig. S11. Capillary samples of $(C_6F_6)_3$:$(C_4H_5N)_4$ and C_6F_6:C_5H_5N S32

Fig. S12. Labelled atoms in the crystal structure of C_6F_6:C_5H_5N S33

Fig. S13. Electron density and Hirshfeld surface for C_6F_6:C_5H_5N S34

Fig. S14. VT-PXRD data on *p*-$C_6H_4Me_2$:C_6F_5H S35

Fig. S15. Volume of *p*-$C_6H_4Me_2$:C_6F_5H as a function of temperature S36

Fig. S16. DSC data on *p*-$C_6H_4Me_2$:C_6F_5H S37

Fig. S17. Labelled atoms in the crystal structure of $(C_6F_6)_3$:$(C_4H_5N)_4$ in phase I S38

Fig. S18. Labelled atoms in the crystal structure of $(C_6F_6)_3$:$(C_4H_5N)_4$ in phase II S39

S1. Sample preparation

Co−crystal of C_4H_5N and C_6F_6:

1.86 g of hexafluorobenzene (C_6F_6, 0.01 mol, Sigma Aldrich Ltd., H8706, M.W. = 186.05, 99% purity) and 0.67 g of pyrrole (C_4H_5N, 0.01 mol, Sigma Aldrich Ltd. 131709, M.W. = 67.09, 98% purity) were added to a glass sample vial giving a clear, homogeneous equimolar liquid mixture. Subsequently, a second sample was prepared for DSC in the correct 3:4 ratio to match the co-crystal structure.

Co−crystal of C_5H_5N and C_6F_6:

1.86 g of hexafluorobenzene (C_6F_6, 0.01 mol, Sigma Aldrich Ltd., H8706, M.W. = 186.05, 99% purity) and 0.79 g of pyridine (C_5H_5N, 0.01 mol, Sigma Aldrich Ltd., 360570, M.W. = 79.10, ≥99% purity) were added to a glass sample vial giving a clear, homogeneous liquid mixture.

Co−crystal of C_6F_5H and *p*-C_8H_{10}:

1.11 g of pentafluorobenzene (C_6F_5H, 0.01 mol, Fluorochem, 002836, Batch FCB114752, M.W. = 168.06, 99% purity) and 1.23 g of *p*-xylene (C_8H_{10}, 0.01 mol, Sigma-Aldrich, 95682-2.5 L, MW = 106.17, GC grade ≥ 99%) were added to a glass sample vial giving a clear, homogeneous liquid mixture.

S2. Single-crystal X-ray diffraction (SXD) experiments

X−ray diffraction data on crystals of $(C_6F_6)_3$:$(C_4H_5N)_4$ and C_6F_6:C_5H_5N were obtained using an Agilent Oxford Diffraction SuperNova diffractometer equipped with microfocus X−ray sources, and upgraded with a Rigaku HyPix Arc-100 detector (Fig. S1). X−ray diffraction data on *p*-$C_6H_4Me_2$:C_6F_5H were obtained on the Agilent Oxford Diffraction SuperNova diffractometer equipped using the older Atlas CCD detector, shortly before the installation of the new HyPix one. All X-ray diffraction data were obtained with Cu Kα radiation (λ = 1.54184 Å).

S2.1. Crystal growing from equimolar mixture of C_4H_5N and C_6F_6

The flame-sealed capillary containing C_6F_6:C_4H_5N was mounted on the diffractometer and cooled rapidly *in situ* to 230 K resulting in the formation of numerous white crystals. The temperature was then increased to 260 K, (just below the melting point at 266 K) in order to encourage annealing of single crystals present. The sample was held at 260 K for 2 hours before cooling to 254 K for data acquisition. Post structure solution, it was found that the co−crystal from an equimolar mixture of C_4H_5N and C_6F_6 did not crystallise in a 1:1 molar ratio, but rather in a 3:4 ratio, which explained the difficulties encountered when trying to grow single crystals of this material. The use of the wrong ratio resulted in additional unwanted diffraction signal from the excess C_6F_6 present.

S2.2. Crystal growing from equimolar mixture of C_5H_5N and C_6F_6

After mounting on the diffractometer, the capillary containing C_6F_6:C_5H_5N was cooled rapidly *in situ* to 200 K resulting in the formation of numerous white crystals. The temperature was then raised to 230 K, and crystal growth was encouraged by manual slow translation of the capillary sample through the cold N_2 gas stream. Data was initially acquired at 200 K, and then subsequently a full-sphere of data was measured at 150 K.

S2.3. Crystal growing from equimolar mixture of *p*-$C_6H_4Me_2$ and C_6F_5H

After mounting on the diffractometer, the capillary containing *p*-$C_6H_4Me_2$:C_6F_5H was cooled rapidly *in situ* to 200 K resulting in the formation of numerous clear/white crystals. The temperature was then raised to just below the melt at about 255 K, and crystal growth was encouraged by manual slow translation of the capillary sample through the cold N_2 gas stream. A full-sphere of data was initially acquired at 230 K, and then subsequently at 160 K, 200 K, and finally at 120 K. It is noteworthy that the system underwent a phase transition between 160 K and 120 K (later confirmed by DSC), which saw a doubling of the unit cell volume on cooling.

S2.4. Data acquisition, reduction, and structure solution and refinement

Full spheres of X-ray diffraction data were collected to 0.84 Å resolution using 0.5° ω scan frames; 6124 frames at 2 s per frame for $(C_6F_6)_3$:$(C_4H_5N)_4$ and 6496 frames at 0.75 s per frame for C_6F_6:C_5H_5N. This corresponds to a total collection time of under 4 hrs for $(C_6F_6)_3$:$(C_4H_5N)_4$ and about 1 hr 45 min for C_6F_6:C_5H_5N. The Cryojet5® used for the measurements in this study is the original prototype device developed by Oxford Instruments and the Pt−resistance sensor is located in the copper−block heat exchanger and not in the nozzle of the instrument close to the sample. Thus, the temperatures quoted in these SXD experiments should be treated as nominal (despite stability to much better than 0.1 K), especially since the sample mass was considerably larger than for a normal single-crystal experiment. Automatic data reduction was performed using the CrysAlis PRO software package from Rigaku Oxford Diffraction, version 1.171.42.60a for $(C_6F_6)_3$:$(C_4H_5N)_4$ and *p*-$C_6H_4Me_2$:C_6F_5H, and the later version 1.171.42.72a for C_6F_6:C_5H_5N.

The crystal structures of $(C_6F_6)_3$:$(C_4H_5N)_4$ and C_6F_6:C_5H_5N at 150 K were solved using the program SHELXT[1] and refined by least−squares using the refinement program SHELXL[2] within the Olex2 program suite.[3] The positions of all atoms were refined freely, with isotropic displacement parameters used for H atoms. Crystal structures are illustrated with the program Mercury[4] from CCDC with thermal ellipsoids (excluding H atoms) shown at 50% probability, H atoms are shown as fixed radius spheres. CIF files have been uploaded to the Cambridge Crystallographic Data Centre with deposition numbers 2263896 and 2263897.

---

[1] Sheldrick, G. M. (2015). *Acta Cryst.* A**71**, 3–8.
[2] Sheldrick, G. M. (2015). *Acta Cryst.* C**71**, 3–8.
[3] Dolomanov, O.V. *et al*. (2009), *J. Appl. Cryst.* **42**, 339–341.
[4] Macrae, C. F. *et al*. (2008) *J. Appl. Cryst*. **41**, 466−470.

As previously stated, data sets were measured on p-$C_6H_4Me_2$:C_6F_5H at 4 different temperatures: 200 K, 160 K, 230 K, and then 120 K. At 200 K, peak peaking software and automatic indexing identified a triclinic unit cell from around 39% of the visible spots. The high temperature phase I was processed in space group $P\bar{1}$ with a = 6.4137(5) Å, b = 7.2398(5) Å, b = 7.3932(5) Å, α = 107.364(6)°, β = 94.389(6)°, γ = 99.370(6)°, and V = 320.41(4) Å^3. Structure solution using SHELXT[1] suggested that phase I exhibited orientational disorder of the C_6F_5H rings. Cooling to low temperature revealed the existence of phase II. This phase transition was not known in advance but its existence was suspected due to the disorder observed at higher temperatures. Due to instrument availability, SXD measurements were made prior to DSC and PXRD data collection on this sample. At 120 K, a triclinic cell was identified from 45% of the visible spots with a = 8.567 Å, b = 8.779 Å, c = 9.371 Å, α = 79.94°, β = 69.33°, γ = 70.14°, and V = 619.0 Å^3, for which the volume is roughly double that observed at higher temperatures. However, this unit cell does not easily relate to that of phase I. Subsequently, a larger I-centred triclinic unit cell was chosen such that the molecular column axis in both phases is in the same direction along c. The refined parameters for this cell are: a = 10.2296(5) Å, b = 8.7819(5) Å, c = 14.6776(7) Å, α = 106.811(5)°, β = 80.111(4)°, γ = 97.168(4)°, and V = 1239.50(11) Å^3.

The structures of phases I and II of p-$C_6H_4Me_2$:C_6F_5H were refined by least–squares using the refinement program SHELXL[2] within the Olex2 program suite.[3] The positions of all atoms were refined freely, with isotropic displacement parameters used for H atoms. In phase I of p-$C_6H_4Me_2$:C_6F_5H, the centre of mass of both rings sit on a point of inversion within space group $P\bar{1}$. Refinement of the fluorine occupancy for F(1), F(2), and F(3) indicated that the single H atom in C_6F_5H was unevenly distributed across all three possible positions (see Fig. S17). In the least-squares refinement, the total occupancy of the fluorine sites was tightly restrained to 5 per molecule such that the molecular formula is exactly C_6F_5H. It is not possible to locate the single H atom of C_6F_5H in phase I and so its electron density was constrained to be at the same position as the F atoms. In phase I, the methyl group of p-$C_6H_4Me_2$ was assumed to be disordered and is modelled with 6 positions for the three H atoms. In phase II of p-$C_6H_4Me_2$:C_6F_5H, there are two crystallographically-distinct p-$C_6H_4Me_2$ molecules, both of which sit on a point of inversion within space group $I\bar{1}$. The methyl group was modelled as ordered using the AFIX 137 instruction. For C_6F_5H, the site occupancy of all 6 possible F positions were refined: one fluorine showed a low occupancy while the occupancy of sites F(1) and F(4) refined to nearly 100%. The latter were therefore fixed as fully occupied. In this phase, the H atom is mainly located on one site but there is still some residual disorder, possible due to the fact that the measurement was unwittingly made close to the phase-transition temperature. The position of H(2) was refined to be co-axial with the residual fluorine density on this site, but residual H scattering was distributed on to the F(3), F(5) and F(6) sites (see Fig. S18). As with phase I, the total occupancy of the fluorine sites was heavily restrained to maintain the correct molecular formula of C_6F_5H. CIF files have been uploaded to the Cambridge Crystallographic Data Centre with deposition numbers 2287264-2287267.

S3. Powder X-ray diffraction (PXRD) experiments

A small amount of the liquid mixture of p-$C_6H_4Me_2$:C_6F_5H was pipetted into the end of a 1.0 mm X-ray capillary, shaken to the closed end, and then carefully flame-sealed to prevent sample loss, particularly of the more volatile component. The flame-sealed end was checked visually for integrity and the sample was used immediately in the PXRD experiment. Variable temperature powder X-ray diffraction (VT-PXRD) measurements were performed using a Stoe Stadi-P diffractometer equipped with a Cu anode, Ge<111> monochromator, a restricted height collimator to limit axial divergence, a Dectris Mythen 1K detector, and an Oxford Instruments CryojetHT (90-500 K) with an in-house modified sample setup to discourage the formation of ice on the goniometer head at low temperature.

The sample was flash-frozen by placing the capillary directly into the cold jet N_2 gas of the CryojetHT precooled to 100 K to encourage formation of a crystalline powder. The temperature was raised from 100 K to room temperature in 10 K increments. At each temperature, the detector was scanned in 2θ from 2° to 60° in steps of 0.5° at 10 s per step, a complete scan lasting approx. 30 min; each 10 K temperature change took approx. 7-10 min and the sample was kept at the set temperature for 5 min before starting the next scan.

The primary purpose of our VT-PXRD analysis is the identification of phase transitions and the determination of lattice parameters and unit cell volume as a function of temperature as data collected for this purpose are not of sufficient statistical quality for structure analysis using the Rietveld method. Le Bail whole pattern fitting[5] using the program Rietica[6] (version 1.7.7) was used to refine the cell parameters from the data shown in Fig. S14. The results are tabulated in Table S5. It is noteworthy that large errors on the lattice parameters are likely to be obtained when the crystal system is triclinic, when a second impurity phase is present, and when the sample is not an ideal powder due to large crystallites as observed here; consequently, on several occasions inaccurate lattice parameters were obtained due to the presence of false minima and these fits had to be repeated. The variation in molecular volume (obtained by dividing the unit-cell volume by the number of molecules per cell, Z) with temperature is plotted in Fig. S15.

S4. Differential scanning calorimetry (DSC) experiments

37.4 mg of C_6F_6:C_4H_5N (prepared as 0.01 mol to 0.013 mol, 3:4 ratio) was loaded into an Al pan, covered with an Al lid, and crimped. The sample pan was then loaded into a PerkinElmer DSC8000 calorimeter at +20 °C. A helium purge gas was used (40 mL min^{-1}). The sample was initially warmed to 30 °C (heating rate 10 °C min^{-1}) and held at this temperature for 1 min. It was then cooled to −180 °C at 10 °C min^{-1}, held at low temperature for 1 min, and then heated back to 30 °C. The data is shown in Fig. S10. Subsequent data analysis to determine both peak maxima and peak area used the Pyris Thermal Analysis software (version 11.1.1.0492) from PerkinElmer. The data showed a sharp single exothermic peak at 240.2 K on cooling due to freezing of the liquid and a broader endothermic peak at 266.4 K on the return heating cycle due to melting of the solid. The enthalpy of fusion is

[5] A. Le Bail, *Powder Diffr.*, 2005, **20**, 316–326.
[6] *http://www.ccp14.ac.uk/tutorial/lhpm-rietica/index.html* ; *http://www.rietica.org/* .

48 kJ mol^{-1}. It is noteworthy that an additional small exothermic peak observed at 233.3 K on cooling and the equivalent endothermic peak observed at 246.8 K on heating are attributed to a slight excess of C_4H_5N in the mixture from a handling loss of the more highly volatile C_6F_6.

As there was no suggestion of a phase transition in the SXD experiments on C_6F_6:C_5H_5N, no DSC data was obtained on this system. DSC data was obtained on *p*-$C_6H_4Me_2$:C_6F_5H using the same protocol as for C_6F_6:C_4H_5N. The data is shown in Fig. S16. The mass of sample used was 18.930 mg with a similar scan to that used for C_6F_6:C_5H_5N, but with the final temperature limited to 20 °C. The DSC shows a sharp exothermic peak at 248.7 K on cooling due to freezing of the liquid and a broader endothermic peak at 256.5 K on the return heating cycle due to melting of the solid. The enthalpy of fusion is 20 kJ mol^{-1}. In addition, and in agreement with VT-PXRD, a solid-state phase transition was observed at low temperature. The enthalpy change on heating was 0.3 kJ mol^{-1}. On cooling, the observed transition temperature was at 125.8 K while on heating the transition was at 128.7 K.

S5. Hirshfeld surface calculations

Hirshfeld and electron density surfaces were calculated in the CrystalExplorer[7] software by selecting the desired fragment and using the "Generate Surface" function. The software can generate various surfaces including Hirshfeld and electron density. The surface is visualised in terms of the normalized contact distance (d_{norm}) which is based on both contact distances between nearest atoms present inside (d_i) and outside (d_e) the chosen fragment. For the surfaces produced for Figs. S9 and S13, it was necessary to set the pixel resolution to the highest setting available. Surfaces were not generated for on *p*-$C_6H_4Me_2$:C_6F_5H due to the disorder seen in both phases.

[7] Spackman, P. R. *et al*. (2021). *J. Appl. Cryst*. **54**, 1006–1011.

Table S1a Crystal data and structure refinement for $(C_6F_6)_3{:}(C_4H_5N)_4$ at 150 K.

Identification code	exp_197
Empirical formula	$C_{34}H_{20}F_{18}N_4$
Formula weight	826.54
Temperature / K	150.0
Crystal system	monoclinic
Space group	$P2_1/n$
a / Å	12.6847(3)
b / Å	5.8648(2)
c / Å	23.2236(5)
α / °	90
β / °	93.284(2)
γ / °	90
Volume / Å^3	1724.84(8)
Z	2
ρ_{calc} / g cm^{-3}	1.591
μ / mm^{-1}	1.476
F(000)	828.0
Crystal size / mm^3	0.943 × 0.436 × 0.415
Radiation	Cu Kα (λ = 1.54184 Å)
2θ range for data collection / °	7.626 to 156.186
Index ranges	$-15 \le h \le 15$, $-7 \le k \le 6$, $-29 \le l \le 29$
Reflections collected	34525
Independent reflections	3554 [R_{int} = 0.1005, R_{sigma} = 0.0317]
Data/restraints/parameters	3554/0/293
Goodness-of-fit on F^2	1.051
Final R indexes [$I \ge 2\sigma(I)$]	R_1 = 0.0605, wR_2 = 0.1594
Final R indexes [all data]	R_1 = 0.0701, wR_2 = 0.1669
Largest diff. peak/hole / e Å^{-3}	0.31/−0.26

Table S1b Fractional atomic coordinates and equivalent isotropic displacement parameters for $(C_6F_6)_3$:$(C_4H_5N)_4$ at 150 K. U_{eq} is defined as ⅓ of the trace of the orthogonalised U_{ij} tensor.

Atom	*x*	*y*	*z*	*U*(eq) / Å²
F(1)	0.3034(13)	0.1948(4)	0.2653(8)	0.0879(6)
F(2)	0.4205(16)	−0.0817(3)	0.2000(8)	0.0877(6)
F(3)	0.6211(16)	0.0365(4)	0.1796(7)	0.0907(6)
F(4)	0.7039(13)	0.4295(4)	0.2230(7)	0.0834(6)
F(5)	0.5862(14)	0.7063(3)	0.2863(7)	0.0766(5)
F(6)	0.3876(14)	0.5882(3)	0.3087(7)	0.0818(5)
C(1)	0.4018(19)	0.2518(5)	0.2547(10)	0.0575(6)
C(2)	0.4617(2)	0.1128(4)	0.2220(10)	0.0596(6)
C(3)	0.5634(2)	0.1709(5)	0.2112(9)	0.0590(6)
C(4)	0.6054(19)	0.3712(5)	0.2332(10)	0.0558(6)
C(5)	0.5451(2)	0.5104(4)	0.2655(10)	0.0540(6)
C(6)	0.4447(2)	0.4516(4)	0.2764(10)	0.0555(6)
F(7)	0.3912(11)	0.1115(2)	−0.0254(7)	0.0648(4)
F(8)	0.4674(12)	0.1905(3)	0.0840(6)	0.0651(4)
F(9)	0.5755(13)	0.5803(3)	0.1098(6)	0.0706(5)
C(7)	0.4451(17)	0.3034(4)	−0.0128(10)	0.0487(5)
C(8)	0.4833(17)	0.3438(4)	0.0427(10)	0.0491(5)
C(9)	0.5383(17)	0.5412(4)	0.0556(9)	0.0498(5)
N(1)	0.4935(18)	0.2384(4)	0.4138(9)	0.0613(6)
C(10)	0.5463(2)	0.0807(5)	0.3854(11)	0.0643(7)
C(11)	0.6423(3)	0.1660(6)	0.3762(11)	0.0722(9)
C(12)	0.6476(2)	0.3863(6)	0.4000(11)	0.0657(7)
C(13)	0.5544(2)	0.4251(5)	0.4227(11)	0.0601(6)
N(2)	0.2020(17)	0.4650(5)	0.0143(11)	0.0654(6)
C(14)	0.2079(2)	0.3510(5)	0.0647(15)	0.0702(8)
C(15)	0.2506(2)	0.4895(7)	0.1052(14)	0.0738(9)
C(16)	0.2730(2)	0.6947(6)	0.0791(15)	0.0728(8)
C(17)	0.2425(2)	0.6757(5)	0.0232(14)	0.0663(7)
H(1)	0.430(2)	0.228(5)	0.4200(12)	0.0600(8)
H(10)	0.512(3)	−0.058(7)	0.3738(18)	0.1060(13)
H(11)	0.689(3)	0.099(6)	0.3596(16)	0.0890(11)
H(12)	0.703(3)	0.478(6)	0.3996(13)	0.0720(9)
H(13)	0.529(2)	0.549(6)	0.4422(14)	0.0730(9)
H(2)	0.176(3)	0.402(6)	−0.0152(16)	0.0860(11)
H(14)	0.180(3)	0.204(7)	0.0670(16)	0.0970(11)
H(15)	0.267(3)	0.456(7)	0.139(2)	0.1100(13)
H(16)	0.310(3)	0.823(8)	0.0934(19)	0.1220(14)
H(17)	0.249(3)	0.772(7)	−0.0048(16)	0.0940(11)

Table S1c Anisotropic displacement parameters for $(C_6F_6)_3$:$(C_4H_5N)_4$ at 150 K. The anisotropic displacement factor exponent has the form: $-2\pi^2[h^2a^{*2}U_{11}+2hka^*b^*U_{12}+\ldots]$.

Atom	U_{11} / Å^2	U_{22} / Å^2	U_{33} / Å^2	U_{23} / Å^2	U_{13} / Å^2	U_{12} / Å^2
F(1)	0.0673(9)	0.1071(15)	0.0883(12)	0.0168(11)	−0.0048(8)	−0.0165(9)
F(2)	0.1241(15)	0.0517(10)	0.0825(11)	−0.0069(8)	−0.0372(10)	−0.0105(9)
F(3)	0.1123(13)	0.0970(14)	0.0617(9)	−0.0190(9)	−0.0047(9)	0.0431(11)
F(4)	0.0669(9)	0.1069(15)	0.0765(11)	0.0189(10)	0.0048(8)	−0.0073(9)
F(5)	0.1000(12)	0.0478(9)	0.0790(10)	0.0005(7)	−0.0211(9)	−0.0111(8)
F(6)	0.0872(11)	0.0889(13)	0.0686(10)	−0.0156(9)	−0.0013(8)	0.0317(9)
C(1)	0.0590(13)	0.0613(16)	0.0506(12)	0.0112(11)	−0.0093(10)	−0.0036(11)
C(2)	0.0855(17)	0.0407(13)	0.0496(12)	0.0021(10)	−0.0223(11)	−0.0041(12)
C(3)	0.0771(16)	0.0597(16)	0.0391(11)	−0.0011(10)	−0.0059(10)	0.0187(12)
C(4)	0.0613(13)	0.0613(16)	0.0442(11)	0.0129(10)	−0.0025(9)	−0.0006(11)
C(5)	0.0704(14)	0.0384(12)	0.0514(12)	0.0045(9)	−0.0126(10)	−0.0004(11)
C(6)	0.0684(14)	0.0524(15)	0.0446(11)	0.0008(10)	−0.0054(10)	0.0134(11)
F(7)	0.0651(8)	0.0451(8)	0.0834(10)	−0.0050(7)	−0.0029(7)	−0.0062(6)
F(8)	0.0731(9)	0.0592(9)	0.0635(8)	0.0242(7)	0.0079(7)	0.0061(7)
F(9)	0.0846(10)	0.0779(11)	0.0475(7)	−0.0037(7)	−0.0126(7)	0.0014(8)
C(7)	0.0480(11)	0.0389(12)	0.0587(12)	−0.0005(9)	−0.0007(9)	0.0013(9)
C(8)	0.0508(11)	0.0464(13)	0.0502(11)	0.0111(9)	0.0048(9)	0.0060(9)
C(9)	0.0516(11)	0.0537(14)	0.0434(10)	−0.0001(9)	−0.0035(8)	0.0069(10)
N(1)	0.0559(12)	0.0640(15)	0.0638(13)	0.0071(10)	0.0031(9)	−0.0026(10)
C(10)	0.0847(18)	0.0541(16)	0.0527(13)	0.0018(11)	−0.0084(12)	0.0021(14)
C(11)	0.0751(17)	0.0970(2)	0.0439(13)	−0.0016(13)	0.0008(12)	0.0295(17)
C(12)	0.0632(14)	0.0800(2)	0.0527(13)	0.0112(13)	−0.0062(11)	−0.0175(14)
C(13)	0.0735(16)	0.0516(16)	0.0547(13)	0.0004(11)	−0.0003(11)	0.0019(12)
N(2)	0.0577(11)	0.0783(17)	0.0594(13)	−0.0208(12)	−0.0026(9)	0.0002(11)
C(14)	0.0654(15)	0.0438(16)	0.1030(2)	0.0052(15)	0.0204(15)	0.0041(12)
C(15)	0.0756(17)	0.0890(2)	0.0567(15)	0.0052(15)	−0.0013(13)	0.0313(16)
C(16)	0.0574(14)	0.0611(19)	0.0980(2)	−0.0273(16)	−0.0111(14)	0.0048(12)
C(17)	0.0567(13)	0.0612(18)	0.0816(19)	0.0202(15)	0.0092(13)	0.0033(12)

Table S1d Selected bond lengths for $(C_6F_6)_3$:$(C_4H_5N)_4$ at 150 K.

Atom — Atom	Length / Å	Atom — Atom	Length / Å
F(1) — C(1)	1.329(3)	C(7) — C(8)	1.371(3)
F(2) — C(2)	1.343(3)	C(7) — C(9)^1	1.372(3)
F(3) — C(3)	1.326(3)	C(8) — C(9)	1.376(3)
F(4) — C(4)	1.330(3)	C(9) — C(7)^1	1.372(3)
F(5) — C(5)	1.340(3)	N(1) — C(10)	1.338(4)
F(6) — C(6)	1.338(3)	N(1) — C(13)	1.349(4)
C(1) — C(2)	1.373(4)	C(10) — C(11)	1.345(5)
C(1) — C(6)	1.376(4)	C(11) — C(12)	1.405(5)
C(2) — C(3)	1.371(4)	C(12) — C(13)	1.341(4)
C(3) — C(4)	1.376(4)	N(2) — C(14)	1.347(4)
C(4) — C(5)	1.371(4)	N(2) — C(17)	1.349(4)
C(5) — C(6)	1.357(4)	C(14) — C(15)	1.333(5)
F(7) — C(7)	1.340(3)	C(15) — C(16)	1.384(5)
F(8) — C(8)	1.340(2)	C(16) — C(17)	1.339(4)
F(9) — C(9)	1.339(3)		

^{1} $1-x, 1-y, -z$

Table S1e Selected bond angles for $(C_6F_6)_3$:$(C_4H_5N)_4$ at 150 K.

Atom — Atom — Atom	Angle / °	Atom — Atom — Atom	Angle / °
F(1) — C(1) — C(2)	120.6(3)	F(7) — C(7) — C(9)^1	119.8(2)
F(1) — C(1) — C(6)	120.2(3)	C(8) — C(7) — C(9)^1	120.3(2)
C(2) — C(1) — C(6)	119.3(2)	F(8) — C(8) — C(7)	119.9(2)
F(2) — C(2) — C(1)	119.9(3)	F(8) — C(8) — C(9)	120.2(2)
F(2) — C(2) — C(3)	119.4(3)	C(7) — C(8) — C(9)	119.9(2)
C(3) — C(2) — C(1)	120.6(2)	F(9) — C(9) — C(7)^1	120.4(2)
F(3) — C(3) — C(2)	120.6(3)	F(9) — C(9) — C(8)	119.7(2)
F(3) — C(3) — C(4)	119.9(3)	C(7)^1 — C(9) — C(8)	119.9(2)
C(2) — C(3) — C(4)	119.5(2)	C(10) — N(1) — C(13)	109.8(2)
F(4) — C(4) — C(3)	120.0(2)	N(1) — C(10) — C(11)	107.7(3)
F(4) — C(4) — C(5)	120.3(2)	C(10) — C(11) — C(12)	107.7(3)
C(5) — C(4) — C(3)	119.7(2)	C(13) — C(12) — C(11)	106.7(3)
F(5) — C(5) — C(4)	119.3(2)	C(12) — C(13) — N(1)	108.1(3)
F(5) — C(5) — C(6)	120.0(2)	C(14) — N(2) — C(17)	108.7(3)
C(6) — C(5) — C(4)	120.6(2)	C(15) — C(14) — N(2)	108.1(3)
F(6) — C(6) — C(1)	120.0(2)	C(14) — C(15) — C(16)	107.8(3)
F(6) — C(6) — C(5)	119.8(2)	C(17) — C(16) — C(15)	107.3(3)
C(5) — C(6) — C(1)	120.2(2)	C(16) — C(17) — N(2)	108.1(3)
F(7) — C(7) — C(8)	119.9(2)		

^{1} $1-x, 1-y, -z$

Table S2a Crystal data and structure refinement for C_6F_6:C_5H_5N at 150 K.

Identification code	exp_299
Empirical formula	$C_{11}H_5F_6N$
Formula weight	265.16
Temperature / K	150
Crystal system	orthorhombic
Space group	$P2_12_12_1$
a / Å	5.85770(10)
b / Å	10.4534(2)
c / Å	17.3482(3)
α / °	90
β / °	90
γ / °	90
Volume / Å^3	1062.28(3)
Z	4
ρ_{calc} / g cm^{-3}	1.658
μ / mm^{-1}	1.564
F(000)	528.0
Crystal size / mm^3	0.862 × 0.218 × 0.212
Radiation	Cu Kα (λ = 1.54184 Å)
2θ range for data collection / °	9.878 to 159.14
Index ranges	$-7 \le h \le 6$, $-13 \le k \le 13$, $-21 \le l \le 21$
Reflections collected	24728
Independent reflections	2275 [R_{int} = 0.0615, R_{sigma} = 0.0240]
Data/restraints/parameters	2275/0/184
Goodness-of-fit on F^2	1.065
Final R indexes [$I \ge 2\sigma (I)$]	R_1 = 0.0346, wR_2 = 0.0929
Final R indexes [all data]	R_1 = 0.0429, wR_2 = 0.0983
Largest diff. peak/hole / e Å^{-3}	0.13/−0.15
Flack parameter	0.06(7)

Table S2b Fractional atomic coordinates and equivalent isotropic displacement parameters for C_6F_6:C_5H_5N at 150 K. U_{eq} is defined as ⅓ of the trace of the orthogonalised U_{ij} tensor.

Atom	*x*	*y*	*z*	U(eq) / Å^2
F(1)	0.5109(4)	0.4753(16)	0.8221(10)	0.0678(6)
F(2)	0.2028(3)	0.4642(18)	0.7056(10)	0.0672(5)
F(3)	0.2788(3)	0.3088(19)	0.5835(9)	0.0680(6)
F(4)	0.6658(4)	0.1678(17)	0.5764(9)	0.0693(6)
F(5)	0.9736(3)	0.1779(17)	0.6932(11)	0.0650(5)
F(6)	0.8959(3)	0.3323(18)	0.8155(9)	0.0645(5)
C(1)	0.5491(5)	0.3993(2)	0.7614(14)	0.0446(6)
C(2)	0.3932(5)	0.3930(2)	0.7027(14)	0.0452(6)
C(3)	0.4307(5)	0.3146(3)	0.6408(14)	0.0458(6)
C(4)	0.6264(5)	0.2428(2)	0.6371(13)	0.0454(6)
C(5)	0.7833(5)	0.2485(2)	0.6959(14)	0.0446(6)
C(6)	0.7445(5)	0.3271(2)	0.7583(13)	0.0438(6)
N(1)	0.5647(4)	0.6745(2)	0.6118(13)	0.0537(6)
C(7)	0.4135(5)	0.6625(3)	0.5545(16)	0.0483(6)
C(8)	0.4437(5)	0.5830(3)	0.4922(15)	0.0481(6)
C(9)	0.6395(6)	0.5119(3)	0.4879(16)	0.0505(6)
C(10)	0.7985(5)	0.5224(3)	0.5457(18)	0.0516(7)
C(11)	0.7549(6)	0.6045(3)	0.6058(16)	0.0525(7)
H(7)	0.281(6)	0.713(3)	0.5609(19)	0.0690(10)
H(8)	0.325(6)	0.579(3)	0.4552(17)	0.0580(9)
H(9)	0.670(6)	0.459(3)	0.4438(16)	0.0610(9)
H(10)	0.941(6)	0.476(3)	0.5472(18)	0.0600(9)
H(11)	0.863(6)	0.612(3)	0.6501(19)	0.0660(9)

Table S2c Anisotropic displacement parameters for C_6F_6:C_5H_5N at 150 K. The anisotropic displacement factor exponent has the form: $-2\pi^2[h^2a^{*2}U_{11}+2hka^*b^*U_{12}+\ldots]$.

Atom	U_{11} / Å^2	U_{22} / Å^2	U_{33} / Å^2	U_{23} / Å^2	U_{13} / Å^2	U_{12} / Å^2
F(1)	0.0968(16)	0.0557(9)	0.0510(8)	−0.0149(7)	0.0075(9)	0.0084(10)
F(2)	0.0495(11)	0.0666(10)	0.0857(12)	0.0157(9)	0.0070(9)	0.0154(9)
F(3)	0.0723(12)	0.0800(11)	0.0517(8)	0.0157(8)	−0.0230(8)	−0.0248(10)
F(4)	0.1043(15)	0.0590(9)	0.0446(8)	−0.0109(7)	0.0207(10)	−0.0101(11)
F(5)	0.0542(10)	0.0621(10)	0.0788(11)	0.0134(9)	0.0197(9)	0.0156(9)
F(6)	0.0647(11)	0.0762(11)	0.0525(8)	0.0105(8)	−0.0194(8)	−0.0061(10)
C(1)	0.0589(17)	0.0371(11)	0.0380(11)	−0.0018(9)	0.0037(11)	−0.0013(11)
C(2)	0.0385(14)	0.0448(12)	0.0524(13)	0.0092(11)	0.0054(12)	0.0017(11)
C(3)	0.0485(16)	0.0500(13)	0.0389(11)	0.0102(10)	−0.0042(10)	−0.0140(12)
C(4)	0.0619(17)	0.0405(11)	0.0337(10)	−0.0006(9)	0.0119(11)	−0.0093(12)
C(5)	0.0433(15)	0.0422(11)	0.0483(12)	0.0096(10)	0.0114(11)	0.0053(11)
C(6)	0.0469(15)	0.0466(12)	0.0378(11)	0.0062(10)	−0.0060(10)	−0.0045(12)
N(1)	0.0624(16)	0.0486(12)	0.0502(12)	−0.0042(10)	0.0035(11)	0.0005(12)
C(7)	0.0430(15)	0.0456(13)	0.0565(14)	0.0061(11)	0.0057(12)	0.0046(12)
C(8)	0.0478(16)	0.0476(13)	0.0487(13)	0.0053(11)	−0.0045(12)	−0.0074(11)
C(9)	0.0584(18)	0.0429(13)	0.0502(13)	−0.0010(11)	0.0114(13)	−0.0041(12)
C(10)	0.0404(16)	0.0452(13)	0.0691(16)	0.0116(12)	0.0078(13)	0.0027(12)
C(11)	0.0504(17)	0.0524(14)	0.0546(14)	0.0091(12)	−0.0085(13)	−0.0109(13)

Table S2d Selected bond lengths for C_6F_6:C_5H_5N at 150 K.

Atom — Atom	Length / Å	Atom — Atom	Length / Å
F(1) — C(1)	1.337(3)	C(3) — C(4)	1.372(4)
F(2) — C(2)	1.342(3)	C(4) — C(5)	1.374(4)
F(3) — C(3)	1.336(3)	C(5) — C(6)	1.377(3)
F(4) — C(4)	1.334(3)	N(1) — C(7)	1.337(4)
F(5) — C(5)	1.338(3)	N(1) — C(11)	1.338(4)
F(6) — C(6)	1.332(3)	C(7) — C(8)	1.374(4)
C(1) — C(2)	1.370(4)	C(8) — C(9)	1.369(4)
C(1) — C(6)	1.372(4)	C(9) — C(10)	1.374(4)
C(2) — C(3)	1.368(4)	C(10) — C(11)	1.374(4)

Table S2e Selected bond angles for C_6F_6:C_5H_5N at 150 K.

Atom — Atom — Atom	Angle / °	Atom — Atom — Atom	Angle / °
F(1) — C(1) — C(2)	120.2(2)	F(5) — C(5) — C(4)	120.5(2)
F(1) — C(1) — C(6)	119.9(2)	F(5) — C(5) — C(6)	119.6(2)
C(2) — C(1) — C(6)	120.0(2)	C(4) — C(5) — C(6)	119.9(2)
F(2) — C(2) — C(1)	119.9(2)	F(6) — C(6) — C(1)	120.2(2)
F(2) — C(2) — C(3)	119.7(2)	F(6) — C(6) — C(5)	120.0(2)
C(3) — C(2) — C(1)	120.4(2)	C(1) — C(6) — C(5)	119.8(2)
F(3) — C(3) — C(2)	120.3(3)	C(7) — N(1) — C(11)	116.3(2)
F(3) — C(3) — C(4)	119.8(2)	N(1) — C(7) — C(8)	123.8(3)
C(2) — C(3) — C(4)	119.9(2)	C(9) — C(8) — C(7)	118.7(3)
F(4) — C(4) — C(3)	120.2(3)	C(8) — C(9) — C(10)	119.0(3)
F(4) — C(4) — C(5)	119.7(3)	C(11) — C(10) — C(9)	118.5(3)
C(3) — C(4) — C(5)	120.1(2)	N(1) — C(11) — C(10)	123.8(3)

Table S3a Crystal data and structure refinement for phase I of *p*-$C_6H_4Me_2$:C_6F_5H at 160 K.

Identification code	exp_3163
Empirical formula	$C_{14}H_{11}F_5$
Formula weight	274.23
Temperature / K	160
Crystal system	triclinic
Space group	$P\overline{1}$
a / Å	6.3499(4)
b / Å	7.2178(5)
c / Å	7.3804(5)
α / °	108.087(7)
β / °	93.949(6)
γ / °	98.796(6)
Volume / $Å^3$	315.32(4)
Z	1
ρ_{calc} / g cm^{-3}	1.444
μ / mm^{-1}	1.191
F(000)	140.0
Crystal size / mm^3	0.95 × 0.394 × 0.38
Radiation	Cu Kα (λ = 1.54184 Å)
2θ range for data collection / °	14.224 to 144.066
Index ranges	$-7 \le h \le 7$, $-8 \le k \le 8$, $-8 \le l \le 7$
Reflections collected	3974
Independent reflections	1201 [R_{int} = 0.0629, R_{sigma} = 0.0412]
Data/restraints/parameters	1201/61/121
Goodness-of-fit on F^2	1.156
Final R indexes [$I \ge 2\sigma(I)$]	R_1 = 0.0617, wR_2 = 0.1742
Final R indexes [all data]	R_1 = 0.0692, wR_2 = 0.1894
Largest diff. peak/hole / e $Å^{-3}$	0.20/−0.20

Table S3b Fractional atomic coordinates and equivalent isotropic displacement parameters for phase I of *p*-$C_6H_4Me_2$:C_6F_5H at 160 K. U_{eq} is defined as ⅓ of the trace of the orthogonalised U_{ij} tensor.

Atom	*x*	*y*	*z*	*U*(eq) / Å^2	Occupancy
F(1)	-0.4012(2)	-0.1608(3)	0.3161(2)	0.0791(8)	0.972(5)
F(2)	-0.2751(4)	0.2246(4)	0.4284(3)	0.0811(10)	0.697(5)
F(3)	0.1330(4)	0.3918(3)	0.6222(3)	0.0847(9)	0.830(6)
C(1)	-0.2040(4)	-0.0812(4)	0.4065(3)	0.0571(7)	1
C(2)	-0.1368(4)	0.1189(4)	0.4663(3)	0.0568(7)	1
C(3)	0.0655(4)	0.1997(4)	0.5599(3)	0.0598(7)	1
C(4)	0.2014(3)	-0.0151(4)	0.0645(3)	0.0543(7)	1
C(5)	-0.0489(4)	0.1811(4)	0.0152(3)	0.0551(7)	1
C(6)	0.1585(3)	0.1734(3)	0.0824(3)	0.0536(7)	1
C(7)	0.3250(5)	0.3560(5)	0.1681(5)	0.0832(10)	1
H(1)†	$= x_{F(1)}$	$= y_{F(1)}$	$= z_{F(1)}$	$= U_{F(1)}$	$= 1 - O_{F(1)}$
H(2)†	$= x_{F(2)}$	$= y_{F(2)}$	$= z_{F(2)}$	$= U_{F(2)}$	$= 1 - O_{F(2)}$
H(3)†	$= x_{F(3)}$	$= y_{F(3)}$	$= z_{F(3)}$	$= U_{F(3)}$	$= 1 - O_{F(3)}$
H(4)	0.343(5)	-0.016(4)	0.103(4)	0.0720(8)	1
H(5)	-0.076(5)	0.313(5)	0.027(5)	0.0810(9)	1
H(7A)	0.444(10)	0.365(9)	0.091(9)	0.125	½
H(7B)	0.394(13)	0.369(9)	0.296(8)	0.125	½
H(7C)	0.271(7)	0.480(4)	0.186(15)	0.125	½
H(7D)	0.295(10)	0.444(9)	0.291(9)	0.125	½
H(7E)	0.345(13)	0.440(9)	0.086(8)	0.125	½
H(7F)	0.469(5)	0.329(5)	0.196(15)	0.125	½

† The atomic coordinates and atomic displacement parameters of H(1), H(2), and H(3) are tied to those of F(1), F(2), and F(3), respectively. The site occupancies of F(1) plus H(1), F(2) plus H(2), and F(3) plus H(3) are tied to 100% occupancy.

Table S3c Anisotropic displacement parameters for phase I of *p*-$C_6H_4Me_2$:C_6F_5H at 160 K. The anisotropic displacement factor exponent has the form: $-2\pi^2[h^2a^{*2}U_{11}+2hka^*b^*U_{12}+\ldots]$.

Atom	U_{11} / Å^2	U_{22} / Å^2	U_{33} / Å^2	U_{23} / Å^2	U_{13} / Å^2	U_{12} / Å^2
F(1)	0.0541(10)	0.1125(15)	0.0688(11)	0.0262(9)	0.0040(7)	0.0182(8)
F(2)	0.0891(17)	0.1013(19)	0.0777(16)	0.0398(13)	0.0246(11)	0.0626(14)
F(3)	0.0999(16)	0.0670(13)	0.0919(16)	0.0249(10)	0.0192(11)	0.0282(10)
C(1)	0.0494(12)	0.0864(17)	0.0434(11)	0.0243(11)	0.0132(9)	0.0264(11)
C(2)	0.0574(13)	0.0824(16)	0.0481(12)	0.0306(11)	0.0187(9)	0.0401(11)
C(3)	0.0675(15)	0.0680(16)	0.0497(13)	0.0189(11)	0.0193(10)	0.0256(12)
C(4)	0.0450(11)	0.0859(17)	0.0453(12)	0.0280(11)	0.0117(8)	0.0346(11)
C(5)	0.0616(14)	0.0681(14)	0.0521(13)	0.0282(10)	0.0210(10)	0.0373(12)
C(6)	0.0545(13)	0.0696(14)	0.0419(11)	0.0182(10)	0.0162(9)	0.0226(10)
C(7)	0.0791(19)	0.0890(2)	0.0702(18)	0.0127(15)	0.0180(14)	0.0063(16)

Table S3d Selected bond lengths for phase I of *p*-$C_6H_4Me_2$:C_6F_5H at 160 K.

Atom — Atom	Length / Å	Atom — Atom	Length / Å
F(1) — C(1)	1.330(3)	C(3) — C(1)^1	1.378(4)
F(2) — C(2)	1.318(3)	C(4) — C(5)^2	1.360(4)
F(3) — C(3)	1.309(3)	C(4) — C(6)	1.397(3)
C(1) — C(2)	1.361(4)	C(5) — C(4)^2	1.360(4)
C(1) — C(3)^1	1.378(4)	C(5) — C(6)	1.390(3)
C(2) — C(3)	1.366(4)	C(6) — C(7)	1.490(4)

^{1} $-x, -y, 1-z$; ^{2} $-x, -y, -z$

Table S3e Selected bond angles for phase I of *p*-$C_6H_4Me_2$:C_6F_5H at 160 K.

Atom — Atom — Atom	Angle / °	Atom — Atom — Atom	Angle / °
F(1) — C(1) — C(2)	120.1(2)	F(3) — C(3) — C(2)	121.1(2)
F(1) — C(1) — C(3)^1	120.6(3)	C(2) — C(3) — C(1)^1	120.9(3)
C(2) — C(1) — C(3)^1	119.2(2)	C(5)^2 — C(4) — C(6)	121.6(2)
F(2) — C(2) — C(1)	116.6(3)	C(4)^2 — C(5) — C(6)	122.2(2)
F(2) — C(2) — C(3)	123.5(3)	C(4) — C(6) — C(7)	122.1(2)
C(1) — C(2) — C(3)	119.9(2)	C(5) — C(6) — C(4)	116.1(2)
F(3) — C(3) — C(1)^1	117.9(3)	C(5) — C(6) — C(7)	121.8(2)

^{1} $-x, -y, 1-z$; ^{2} $-x, -y, -z$

Table S4a Crystal data and structure refinement for phase II of p-$C_6H_4Me_2$:C_6F_5H at 120 K.

Identification code	exp_3166
Empirical formula	$C_{14}H_{11}F_5$
Formula weight	274.23
Temperature / K	120.5(2)
Crystal system	triclinic
Space group	$I\bar{1}$
a / Å	10.2296(5)
b / Å	8.7819(5)
c / Å	14.6776(7)
α / °	106.811(5)
β / °	80.111(4)
γ / °	97.168(4)
Volume / Å^3	1239.51(12)
Z	4
ρ_{calc} / g cm^{-3}	1.469
μ / mm^{-1}	1.212
F(000)	560.0
Crystal size / mm^3	0.955 × 0.383 × 0.365
Radiation	Cu Kα (λ = 1.54184 Å)
2θ range for data collection / °	10.1 to 145.608
Index ranges	$-12 \le h \le 12$, $-10 \le k \le 10$, $-17 \le l \le 15$
Reflections collected	9202
Independent reflections	2405 [R_{int} = 0.0516, R_{sigma} = 0.0312]
Data/restraints/parameters	2405/8/204
Goodness-of-fit on F^2	1.092
Final R indexes [$I \ge 2\sigma(I)$]	R_1 = 0.0685, wR_2 = 0.2010
Final R indexes [all data]	R_1 = 0.0770, wR_2 = 0.2184
Largest diff. peak/hole / e Å^{-3}	0.78/−0.76

Table S4b Fractional atomic coordinates and equivalent isotropic displacement parameters for phase II of p-$C_6H_4Me_2$:C_6F_5H at 120 K. U_{eq} is defined as ⅓ of the trace of the orthogonalised U_{ij} tensor.

Atom	x	y	z	U(eq) / Å^2	Occupancy
F(1)	0.1222(16)	0.2716(19)	0.3379(11)	0.0556(5)	1
F(2)	0.2523(6)	0.0057(3)	0.2786(3)	0.0770(4)	0.224(6)
F(3)	0.1369(15)	-0.2759(2)	0.1828(12)	0.0527(5)	0.925(2)
F(4)	-0.1166(14)	-0.2922(17)	0.1520(10)	0.0479(4)	1
F(5)	-0.2524(13)	-0.0278(2)	0.2140(11)	0.0493(5)	= $O_{F(3)}$
F(6)	-0.1312(16)	0.2535(2)	0.3092(12)	0.0543(5)	= $O_{F(3)}$
C(1)	0.0641(2)	0.1312(3)	0.2927(15)	0.0383(6)	1
C(2)	0.1338(2)	-0.0021(3)	0.2619(14)	0.0396(6)	1
C(3)	0.0728(2)	-0.1429(3)	0.2151(16)	0.0379(6)	1
C(4)	-0.0578(19)	-0.1525(3)	0.1985(14)	0.0338(5)	1
C(5)	-0.1266(19)	-0.0186(3)	0.2302(15)	0.0356(6)	1
C(6)	-0.0666(2)	0.1242(3)	0.2772(16)	0.0387(6)	1
C(7)	0.1179(2)	-0.0624(3)	0.4974(16)	0.0362(5)	1
C(8)	0.1051(2)	0.0999(3)	0.5364(15)	0.0358(5)	1
C(9)	-0.0133(2)	0.1653(3)	0.5398(15)	0.0348(5)	1
C(10)	-0.0278(3)	0.3408(3)	0.5817(2)	0.0501(7)	1
C(11)	0.1156(2)	0.0835(3)	0.0282(15)	0.0372(6)	1
C(12)	-0.1126(2)	0.0785(3)	0.0144(16)	0.0374(6)	1
C(13)	0.0027(2)	0.1659(3)	0.0441(15)	0.0364(5)	1
C(14)	0.0061(4)	0.3424(3)	0.0901(2)	0.0655(9)	1
H(2)‡	0.2179(3)	0.0035(3)	0.2740(2)	0.047	= 1 − $O_{F(2)}$
H(3)†	= $x_{F(3)}$	= $y_{F(3)}$	= $z_{F(3)}$	0.045	= 1 − $O_{F(3)}$
H(5)†	= $x_{F(5)}$	= $y_{F(5)}$	= $z_{F(5)}$	0.043	= 1 − $O_{F(5)}$
H(6)†	= $x_{F(6)}$	= $y_{F(6)}$	= $z_{F(6)}$	0.046	= 1 − $O_{F(6)}$
H(7)	0.200(3)	-0.105(3)	0.500(2)	0.0500(8)	1
H(8)	0.177(3)	0.173(3)	0.5626(19)	0.0400(7)	1
H(10A)	-0.1046	0.3697	0.5604	0.0750	1
H(10B)	0.0530	0.4010	0.5602	0.0750	1
H(10C)	-0.0413	0.3662	0.6521	0.0750	1
H(11)	0.196(3)	0.142(3)	0.050(2)	0.0520(8)	1
H(12)	-0.186(3)	0.141(4)	0.023(2)	0.0660(9)	1
H(14A)	-0.0133	0.3628	0.1601	0.0980	1
H(14B)	-0.0609	0.3878	0.0675	0.0980	1
H(14C)	0.0946	0.3920	0.0728	0.0980	1

† The atomic coordinates H(3), H(5), and H(6) are tied to those of F(3), F(5), and F(6), respectively. The site occupancies of F(3) plus H(3), F(5) plus H(5), and F(6) plus H(6) are tied to 100% occupancy.
‡ The atomic coordinates of H(2) are restrained such that H(2) lies on the C(2)—F(2) bond.

Table S4c Anisotropic displacement parameters for phase II of p-$C_6H_4Me_2$:C_6F_5H at 120 K. The anisotropic displacement factor exponent has the form: $-2\pi^2[h^2a^{*2}U_{11}+2hka^*b^*U_{12}+\ldots]$.

Atom	U_{11} / Å^2	U_{22} / Å^2	U_{33} / Å^2	U_{23} / Å^2	U_{13} / Å^2	U_{12} / Å^2
F(1)	0.0595(9)	0.0536(9)	0.0465(9)	0.0061(7)	-0.0137(7)	-0.0130(7)
F(2)	0.0230(3)	0.1520(9)	0.0670(5)	0.0530(5)	-0.0110(3)	-0.0110(4)
F(3)	0.0452(8)	0.0551(10)	0.0600(11)	0.0131(8)	-0.0072(7)	0.0190(7)
F(4)	0.0474(8)	0.0471(8)	0.0468(9)	0.0104(6)	-0.0126(6)	-0.0077(6)
F(5)	0.0243(7)	0.0746(11)	0.0545(10)	0.0252(8)	-0.0049(6)	0.0067(6)
F(6)	0.0509(9)	0.0538(10)	0.0572(11)	0.0120(8)	0.0021(7)	0.0226(7)
C(1)	0.0405(12)	0.0473(13)	0.0260(11)	0.0118(9)	-0.0049(9)	-0.0052(9)
C(2)	0.0280(10)	0.0605(15)	0.0337(12)	0.0178(10)	-0.0078(8)	-0.0002(9)
C(3)	0.0340(11)	0.0473(12)	0.0342(12)	0.0140(10)	-0.0010(9)	0.0086(9)
C(4)	0.0331(11)	0.0433(12)	0.0256(10)	0.0122(9)	-0.0051(8)	-0.0025(9)
C(5)	0.0237(9)	0.0568(14)	0.0296(11)	0.0188(10)	-0.0017(8)	0.0024(9)
C(6)	0.0387(12)	0.0439(12)	0.0329(12)	0.0123(10)	0.0037(9)	0.0084(9)
C(7)	0.0267(9)	0.0522(13)	0.0316(11)	0.0145(10)	-0.0031(8)	0.0049(8)
C(8)	0.0257(10)	0.0515(13)	0.0312(11)	0.0150(9)	-0.0056(8)	-0.0044(8)
C(9)	0.0315(10)	0.0459(12)	0.0275(11)	0.0136(9)	0.0000(8)	0.0017(9)
C(10)	0.0526(14)	0.0468(14)	0.0476(15)	0.0124(11)	0.0023(11)	0.0061(11)
C(11)	0.0273(10)	0.0526(14)	0.0321(12)	0.0147(10)	-0.0068(8)	-0.0061(9)
C(12)	0.0280(10)	0.0536(14)	0.0339(12)	0.0169(10)	0.0005(8)	0.0099(9)
C(13)	0.0410(12)	0.0394(11)	0.0262(11)	0.0099(9)	0.0031(9)	0.0031(9)
C(14)	0.0950(2)	0.0398(14)	0.0482(16)	0.0052(12)	0.0125(15)	0.0031(13)

Table S4d Selected bond lengths for phase II of p-$C_6H_4Me_2$:C_6F_5H at 120 K.

Atom — Atom	Length / Å	Atom — Atom	Length / Å
F(1) — C(1)	1.339(3)	C(5) — C(6)	1.370(3)
† H(2) — C(2)	0.90	C(7) — C(8)	1.387(3)
F(3) — C(3)	1.335(3)	C(7) — C(9)^1	1.391(3)
F(4) — C(4)	1.343(2)	C(8) — C(9)	1.392(3)
F(5) — C(5)	1.337(2)	C(9) — C(7)^1	1.391(3)
F(6) — C(6)	1.311(2)	C(9) — C(10)	1.501(3)
C(1) — C(2)	1.369(3)	C(11) — C(12)^2	1.378(3)
C(1) — C(6)	1.385(3)	C(11) — C(13)	1.390(3)
C(2) — C(3)	1.362(3)	C(12) — C(11)^2	1.378(3)
C(3) — C(4)	1.387(3)	C(12) — C(13)	1.388(3)
C(4) — C(5)	1.368(3)	C(13) — C(14)	1.500(3)

^{1} $-x, -y, 1-z$; ^{2} $-x, -y, -z$

† H(2) and F(2) have different positions in the refinement (in contrast to H(3) and F(3), etc.). The H(2)—C(2) bond length was fixed at 0.9 **Å** and the F(2)—C(2) bond length refined to 1.267(7) **Å.**

Table S4e Selected bond angles for phase II of *p*-$C_6H_4Me_2$:C_6F_5H at 120 K.

Atom	— Atom	— Atom	Angle / °	Atom	— Atom	— Atom	Angle / °
F(1) —	C(1)	— C(2)	120.1(2)	C(4) —	C(5)	— C(6)	120.4(2)
F(1) —	C(1)	— C(6)	118.8(2)	F(6) —	C(6)	— C(1)	119.4(2)
C(2) —	C(1)	— C(6)	121.0(2)	F(6) —	C(6)	— C(5)	121.3(2)
H(2)/F(2) —	C(2)	— C(1)	120.2(2)	C(5) —	C(6)	— C(1)	119.26(18)
H(2)/F(2) —	C(2)	— C(3)	120.9(2)	C(8) —	C(7)	— C(9)^1	121.3(2)
C(3) —	C(2)	— C(1)	118.9(2)	C(7) —	C(8)	— C(9)	121.0(2)
F(3) —	C(3)	— C(2)	121.2(2)	C(7)^1 —	C(9)	— C(8)	117.7(2)
F(3) —	C(3)	— C(4)	117.77(19)	C(7)^1 —	C(9)	— C(10)	121.0(2)
C(2) —	C(3)	— C(4)	121.0(2)	C(8) —	C(9)	— C(10)	121.3(2)
F(4) —	C(4)	— C(3)	120.3(2)	C(12)^2 —	C(11)	— C(13)	121.2(2)
F(4) —	C(4)	— C(5)	120.25(19)	C(11)^2 —	C(12)	— C(13)	121.5(2)
C(5) —	C(4)	— C(3)	119.41(19)	C(11) —	C(13)	— C(14)	121.2(2)
F(5) —	C(5)	— C(4)	119.4(2)	C(12) —	C(13)	— C(11)	117.3(2)
F(5) —	C(5)	— C(6)	120.2(2)	C(12) —	C(13)	— C(14)	121.5(2)

^{1} $-x, -y, 1-z$; ^{2} $-x, -y, -z$

Table S5 Lattice parameters and unit cell volume of *p*-$C_6H_4Me_2$:C_6F_5H obtained from Le Bail fits to the VT-PXRD data shown in Fig. S14. Note that the fits for the unit cell of phase II used a unit cell with Z = 4 (as the cell is doubled along *c* and is *I* centred) where phase I is primitive triclinic with Z = 1.

T / K	*a* / Å	*b* / Å	*c* / Å	α / °	β / °	γ / °	V / Å^3
100	10.2147(2)	8.7429(3)	14.6793(6)	106.459(4)	80.476(5)	97.045(2)	1235.9(12)
110	10.2278(2)	8.7573(3)	14.6878(7)	106.585(3)	80.433(5)	97.059(2)	1239.3(12)
120	10.2345(3)	8.7815(3)	14.7228(13)	106.825(5)	80.312(6)	97.146(3)	1244.5(14)
130	6.3086(2)	7.1944(3)	7.3730(7)	108.410(6)	93.673(8)	98.548(3)	311.8(6)
140	6.3199(1)	7.2037(2)	7.3767(6)	108.348(5)	93.781(6)	98.602(3)	312.9(6)
150	6.3324(1)	7.2130(2)	7.3691(10)	108.281(5)	93.831(7)	98.709(3)	313.6(6)
160	6.3466(1)	7.2210(2)	7.3809(7)	108.166(6)	93.979(5)	98.812(3)	315.1(5)
170	6.3614(1)	7.2279(3)	7.3839(10)	108.006(6)	94.052(8)	98.944(3)	316.4(7)
180	6.3769(1)	7.2341(2)	7.3797(10)	107.805(5)	94.094(7)	99.106(3)	317.4(6)
190	6.3939(1)	7.2403(2)	7.3765(11)	107.608(5)	94.179(8)	99.266(3)	318.6(7)
200	6.4133(1)	7.2465(2)	7.3898(12)	107.386(5)	94.360(9)	99.415(3)	320.5(7)
210	6.4336(1)	7.2517(3)	7.3929(5)	107.137(7)	94.500(7)	99.586(3)	322.0(7)
220	6.4547(1)	7.2570(3)	7.3949(8)	106.902(6)	94.654(8)	99.757(3)	323.5(7)
230	6.4784(1)	7.2610(2)	7.3915(8)	106.628(6)	94.791(8)	99.941(3)	324.9(7)
240	6.5033(1)	7.2621(2)	7.3815(4)	106.271(5)	94.876(4)	100.185(2)	326.0(5)

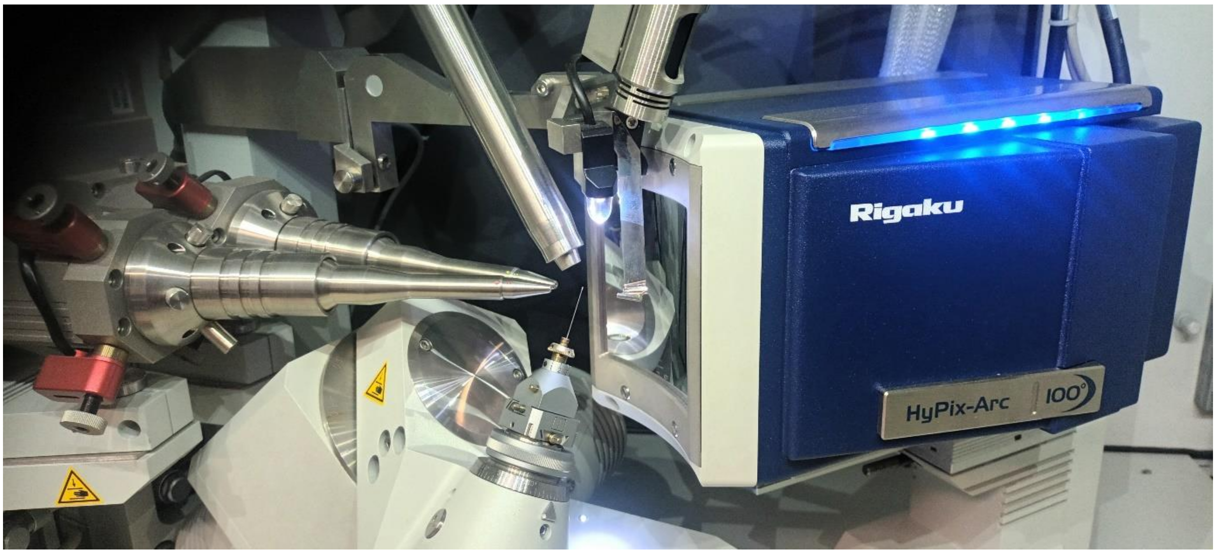

Figure S1 Photograph showing the experimental setup for the X−ray diffraction measurements. Microfocus X−ray sources (Cu and Mo) are seen on the left−hand side, the sample capillary (which is cooled with an Oxford Instruments Cryojet5) is seen mounted in a brass stub on the goniometer head, and the Rigaku HyPix−Arc 100° curved Hybrid Photon Counting (HPC) X−ray detector with "go−faster" blue lights is seen on the right−hand side of the image.

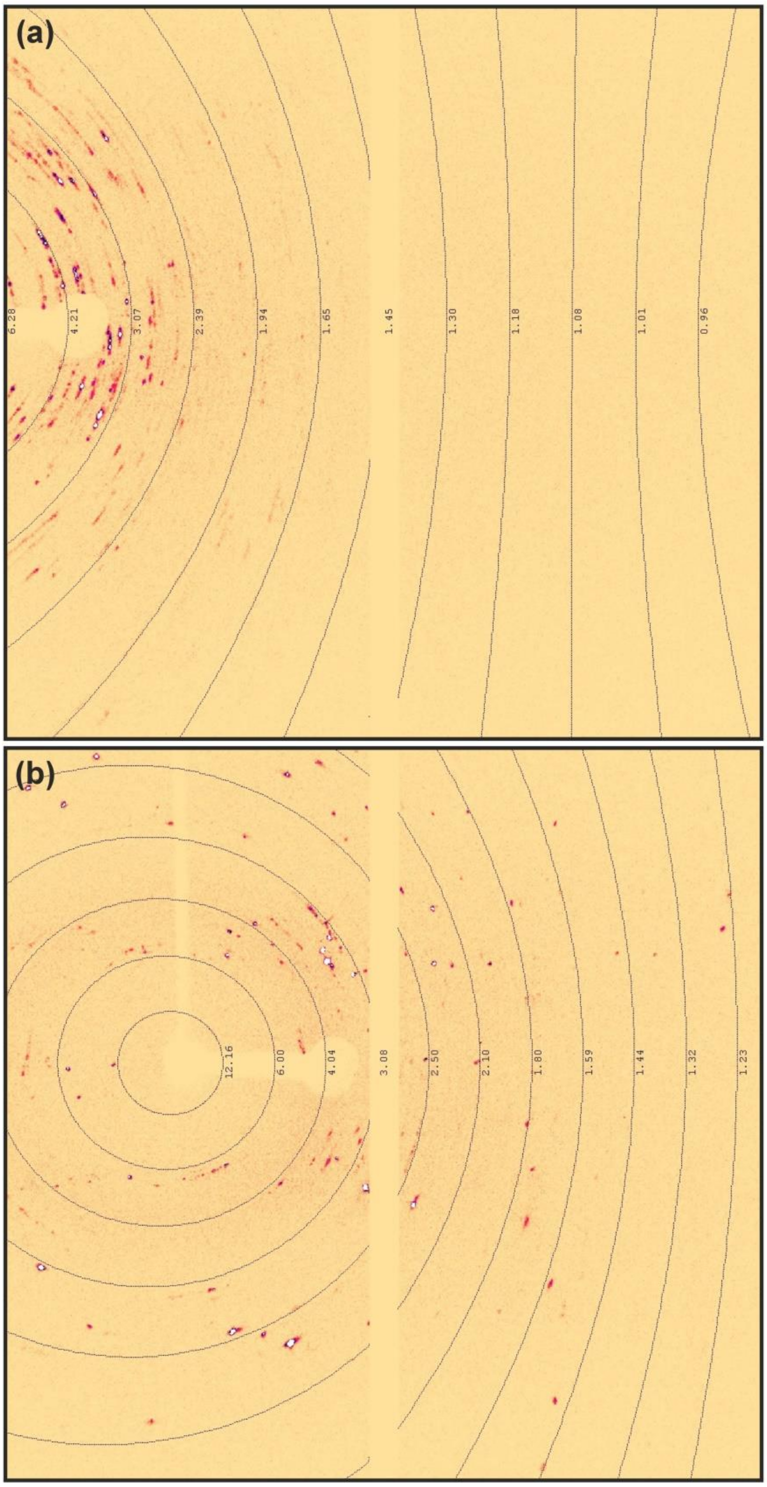

Figure S2 (a) An exemplar frame of raw X-ray data from one crystallisation attempt on $(C_6F_6)_3$:$(C_4H_5N)_4$ demonstrating that the sample in the beam is not suitable for crystal structure solution. There is no sign of diffraction spots at small *d*-spacings (rings of constant *d* with values shown in black). Secondly, the data at low angle shows poorly resolved spots lying on rings, behaviour that is typical of many small crystallites in the beam. (b) By contrast, another exemplar frame of raw X-ray data. The raw data seen here shows clean spots out to small *d*-spacing value even though some spots from different largish crystals are accidentally overlapped.

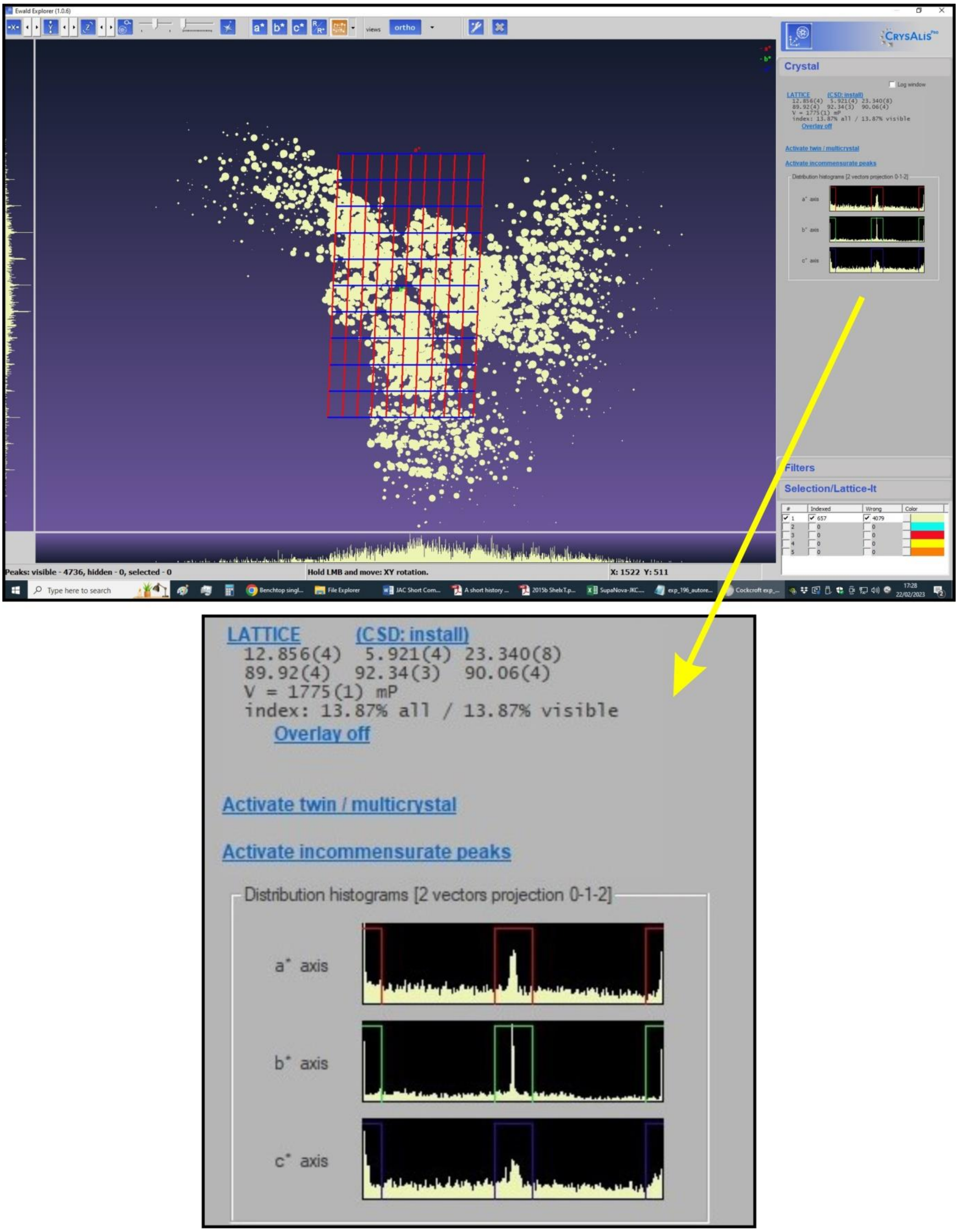

Figure S3 A subset of peak search data with a possible unit cell with the associated lattice for it highlighted in red and blue shown using the Ewald Explorer option within the CrysAlis PRO software from Rigaku. The genuine periodicity in reciprocal space is demonstrated from the sharp peaks seen in the distribution histograms along **a***, **b***, and **c*** directions on the right-hand side, which are shown in an enlarged format in the lower figure.

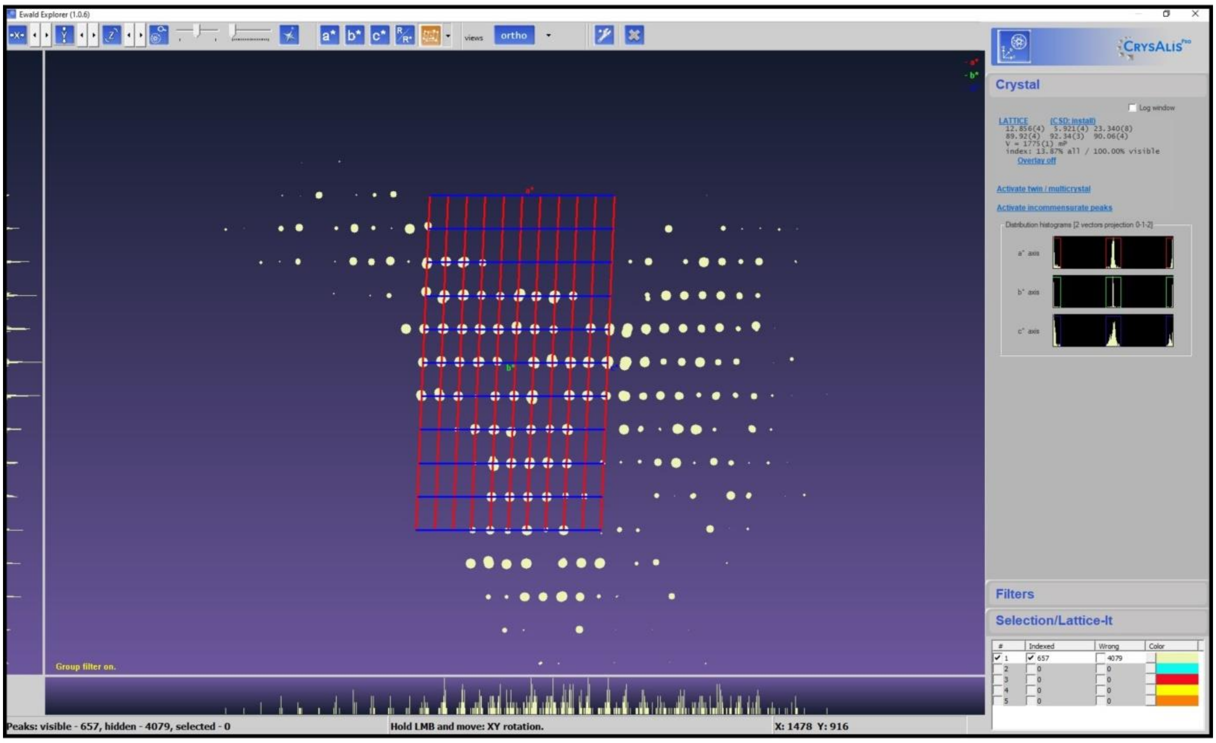

Figure S4 This shows the same data as in Fig. S3 but with the unindexed spots hidden to the viewer. The lattice formed by the diffraction spots is now more evident.

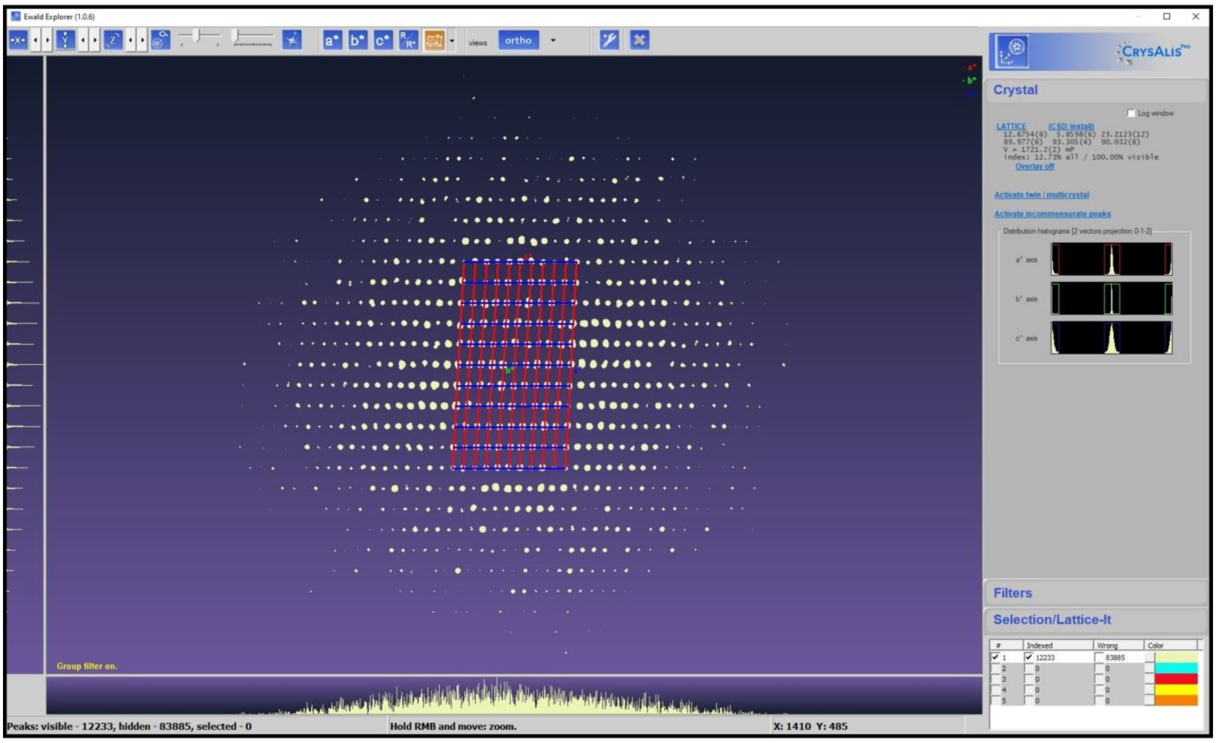

Figure S5 Peak search result from a full-sphere of data with unindexed spots hidden to the viewer. The distribution histograms along **a***, **b***, and **c*** directions on the right-hand side now show a clean and symmetric distribution about the mid-point.

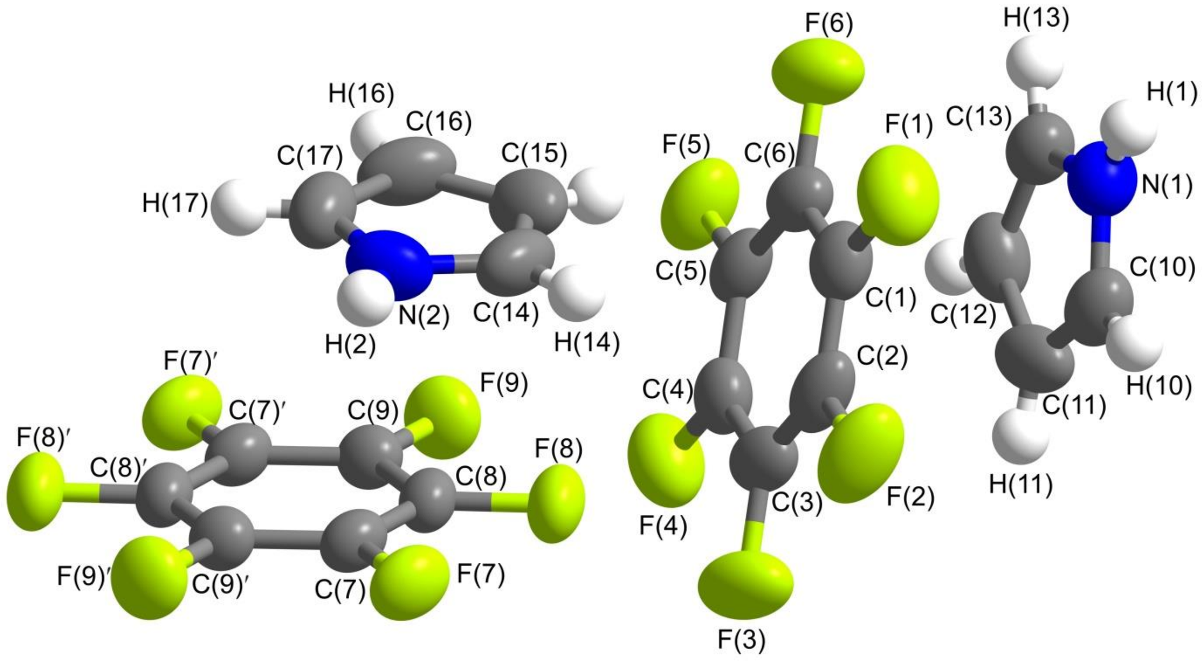

Figure S6 Asymmetric unit from the refined crystal structure of $(C_6F_6)_3$:$(C_4H_5N)_4$ in space group $P2_1/n$ at 150 K showing the crystallographic labelling of the atoms. H atoms are numbered according to the label of the atom to which they are bonded. The labels of a few H atoms are omitted for clarity.

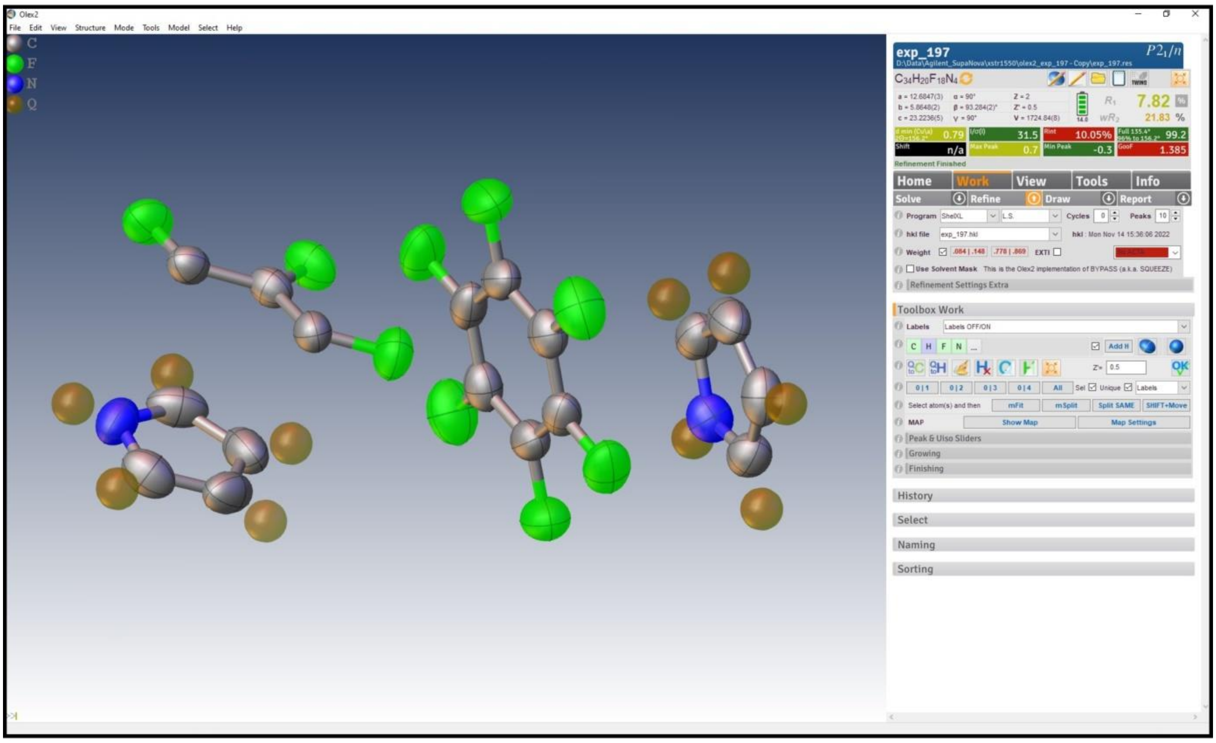

Figure S7 Using the Olex2 program suite[3], a view of the asymmetric unit of the crystal structure of $(C_6F_6)_3{:}(C_4H_5N)_4$ in space group $P2_1/n$ at 150 K showing the refined positions and thermal displacements of the C, N, and F atoms. Positions of highest residual electron density are shown in brown and correspond to the 10 expected positions of the hydrogen atoms.

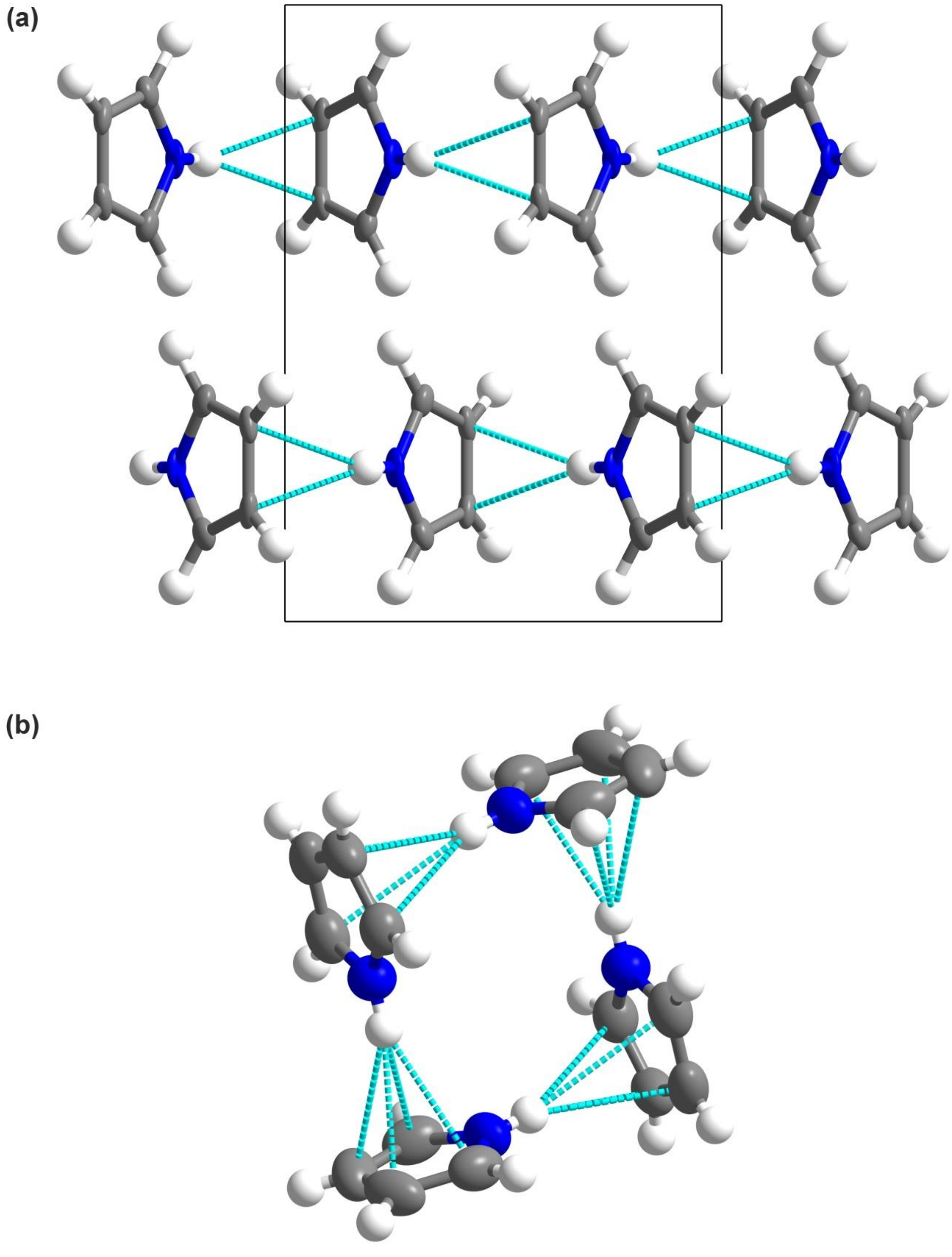

Figure S8 (a) View of the head to tail zig-zag arrangement formed by the molecules in the crystal structure of solid pyrrole as determined by Goddard *et al*. (1997)[8] with close contacts between rings shown as dashed cyan lines; (b) the contrasting tetramer unit formed by the pyrrole molecules in the asymmetric unit of the crystal structure of $(C_6F_6)_3{:}(C_4H_5N)_4$.

---

[8] Goddard, R., Heinemann, O. & Krüger, C. (1997). *Acta Cryst.* C**53**, 1846-1850.

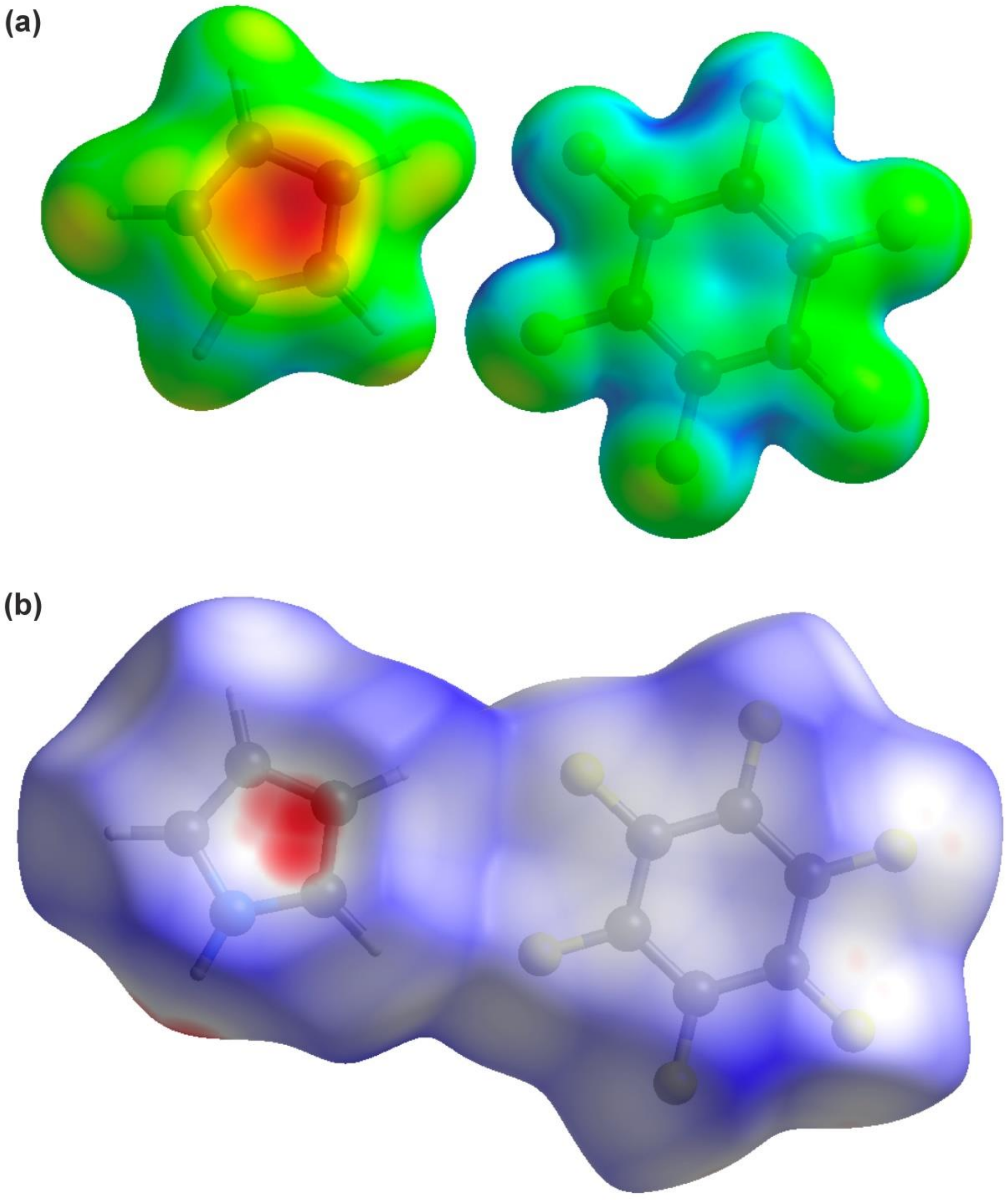

Figure S9 (a) Calculated electron density map for two molecules in $(C_6F_6)_3$:$(C_4H_5N)_4$ with areas of higher electron density presented in red and lower electron density in blue, and green is the neutral point; (b) the Hirshfeld surface for the same two molecules. For the Hirshfeld surface, closest contacts to atoms from other molecules are seen in red and voids between are shown in blue; white is the neutral point.

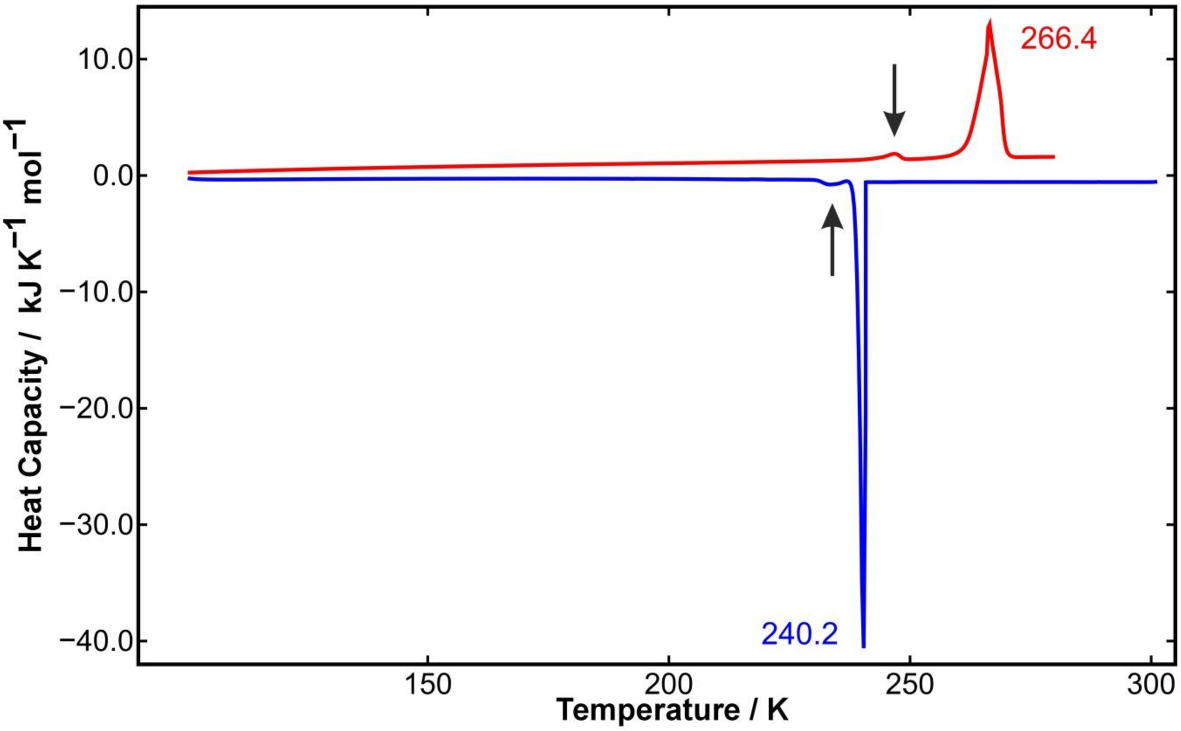

Figure S10 DSC data (shown as "endo up") on $(C_6F_6)_3$:$(C_4H_5N)_4$ prepared as a 3:4 molar ratio sample. The sample exhibits a sharp freezing point at 240.2 K on cooling (blue line) and no other solid−solid phase transitions down to 95 K. On heating (red line), the sample showed a broad melting transition at 266.4 K. The black vertical arrows show a transition due to the presence of a slight excess of pyrrole as a result of the high volatility of C_6F_6.

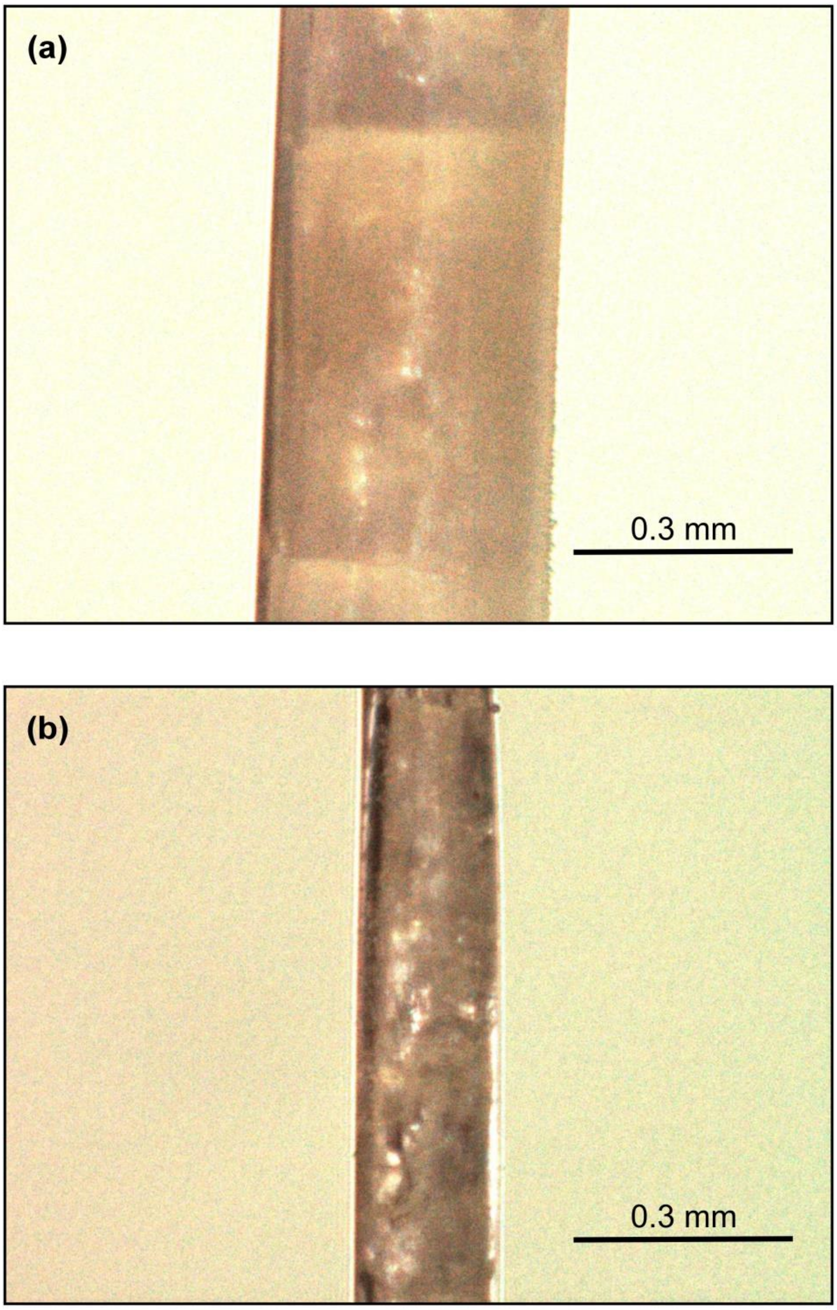

Figure S11 (a) View of the 0.4 mm capillary sample of $(C_6F_6)_3$:$(C_4H_5N)_4$ at 150 K as used in the SXD measurement, and (b) an equivalent view of the 0.2 mm capillary sample of C_6F_6:C_5H_5N at 150 K as used in the later experiment for comparison. Absorption corrections are based on the dimensions of the capillary and *not* the size of some crystal whose size cannot be determined optically. Note that X-ray capillaries are supplied with a nominal size that can vary significantly (*e.g.* ±0.05 mm) from each other within a batch.

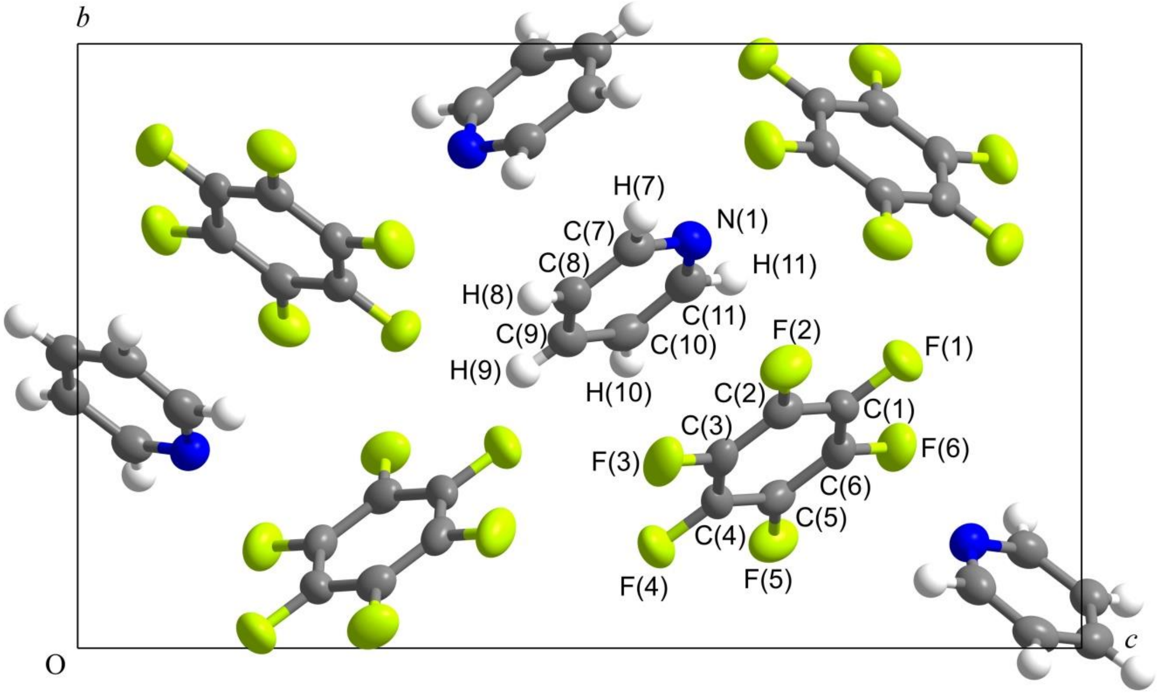

Figure S12 Refined crystal structure of C_6F_6:C_5H_5N in space group $P2_12_12_1$ at 150 K viewed down *a* showing the crystallographic labelling of the atoms. H atoms are numbered according to the label of the atom to which they are bonded.

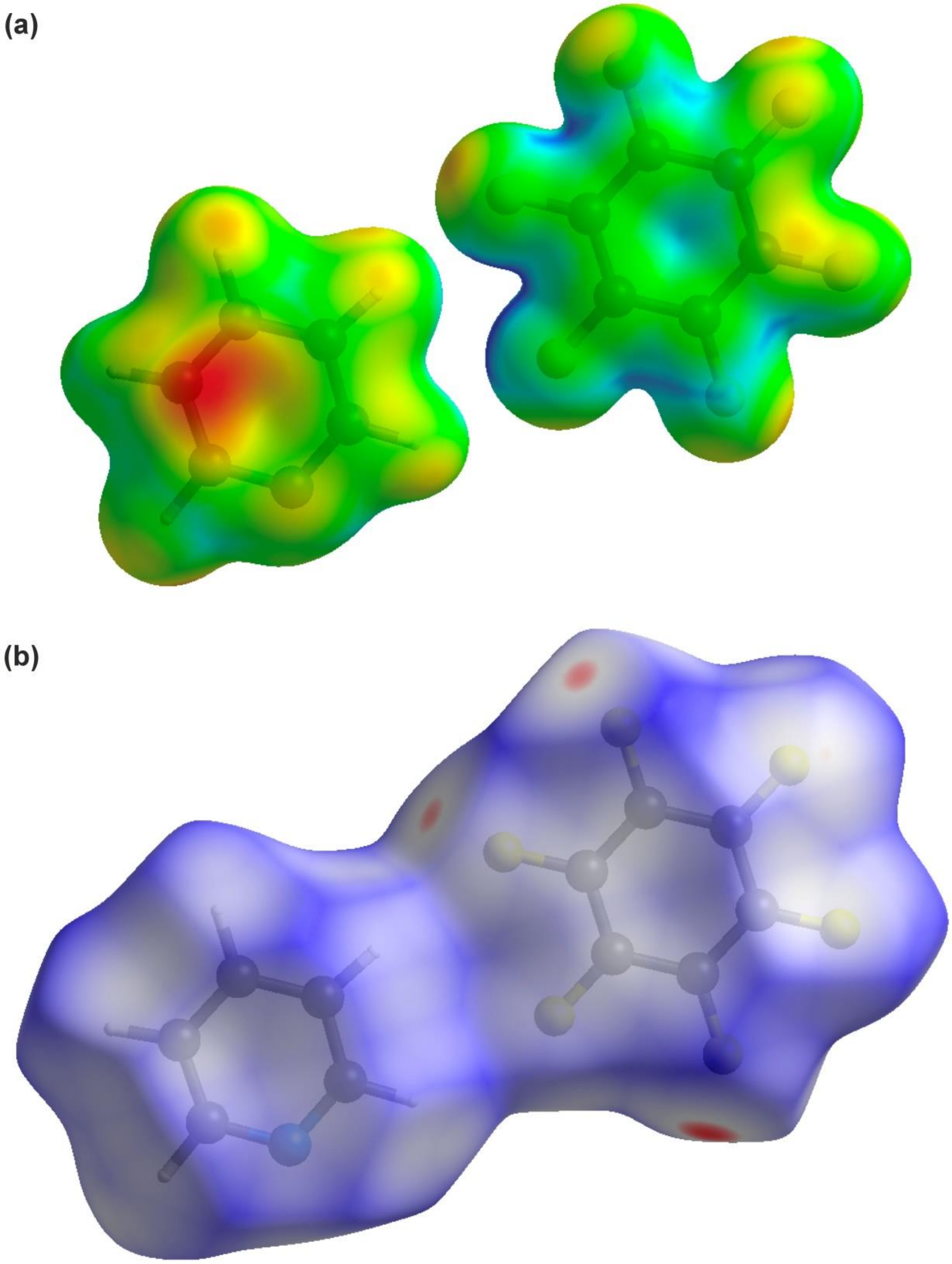

Figure S13 (a) Calculated electron density map for two molecules in C_6F_6:C_5H_5N; (b) the Hirshfeld surface for the same two molecules. The colour schemes are the same as those described in the caption to Fig. S9.

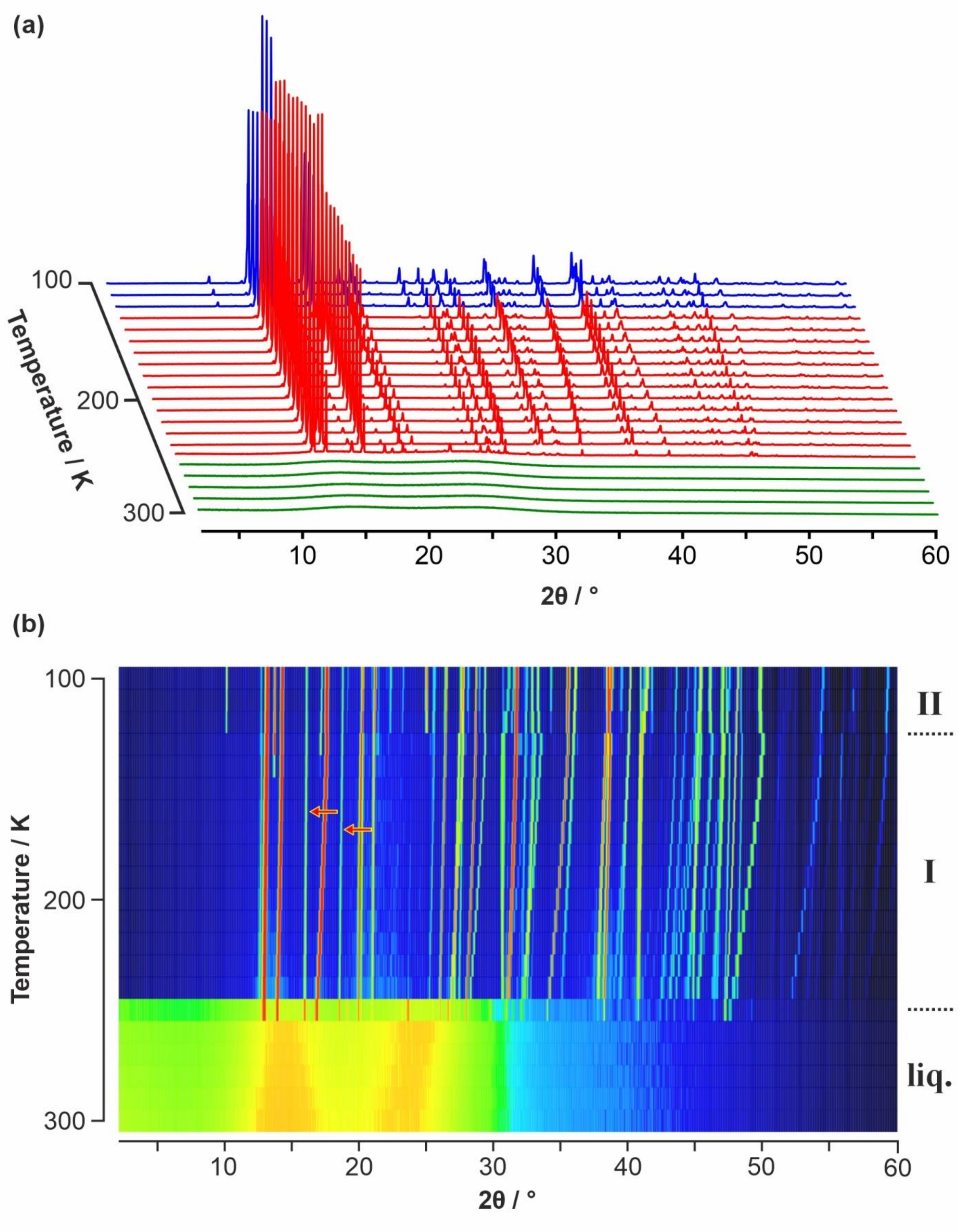

Figure S14 (a) VT-PXRD data on *p*-$C_6H_4Me_2$:C_6F_5H with the data in 10 K steps, phase II is shown in blue, phase I is shown in red, and the liquid state is is shown in green. (b) Same data shown as a colour surface, where the range of intensity is shown as a spectral scale, from low counts in dark blue to high counts in red. The presence of a single solid-state phase transition is clearly seen in the data between 120 K and 130 K. As with $C_6H_{6-n}(CH_3)_n$:C_6F_6 with n = 0, 1, 2, or 3, the liquid state exhibits two characteristic humps, which are visible in the higher temperature patterns. In addition, two weak peaks at about 16.0° and 18.7° (clearly visible in the surface plot and highlighted by the two red arrows) are attributable to a trace excess of *p*-$C_6H_4Me_2$.

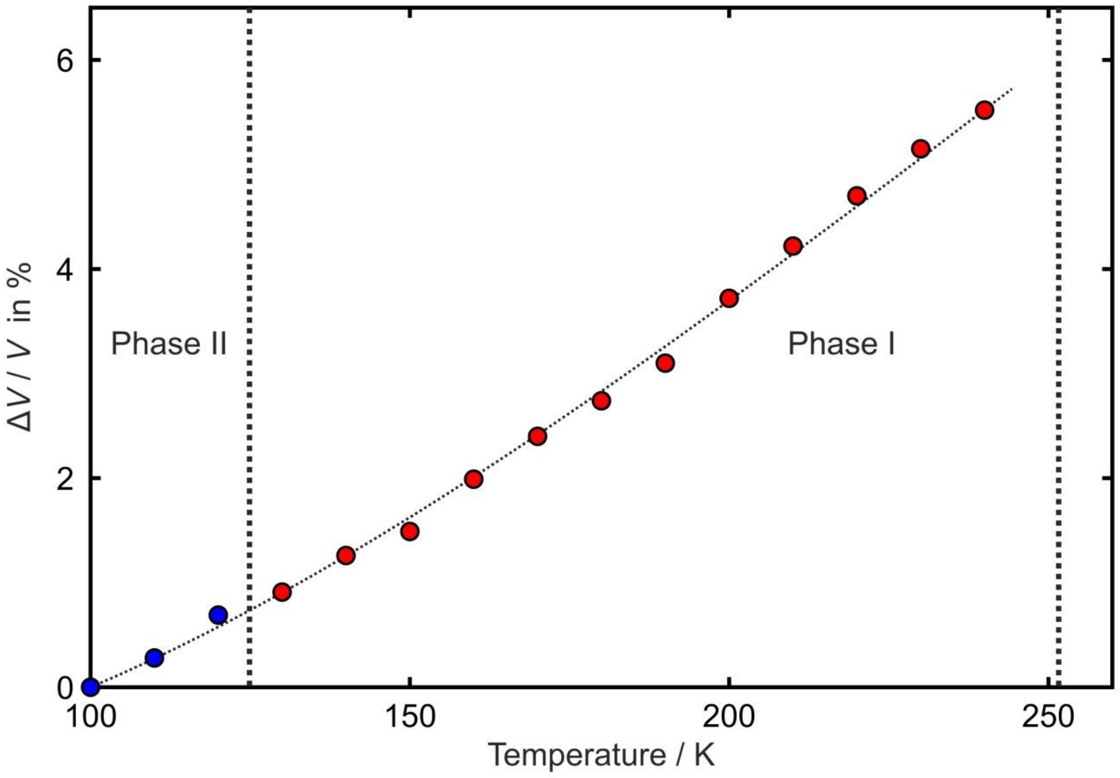

Figure S15 Change in molecular volume relative to 100 K of *p*-$C_6H_4Me_2$:C_6F_5H obtained by Le Bail fits to the VT-PXRD data shown in Fig. S14; the data is presented in Table S5. There is no noticeable change in the volume of *p*-$C_6H_4Me_2$:C_6F_5H on passing through the phase transition, the only slight change is a very small expansion along the column axis *c*. The thermal expansion is mainly due to the increase in the distance between the columns of molecules with increasing temperature as evidenced by the change in the *a* and *b* values given in Table S5.

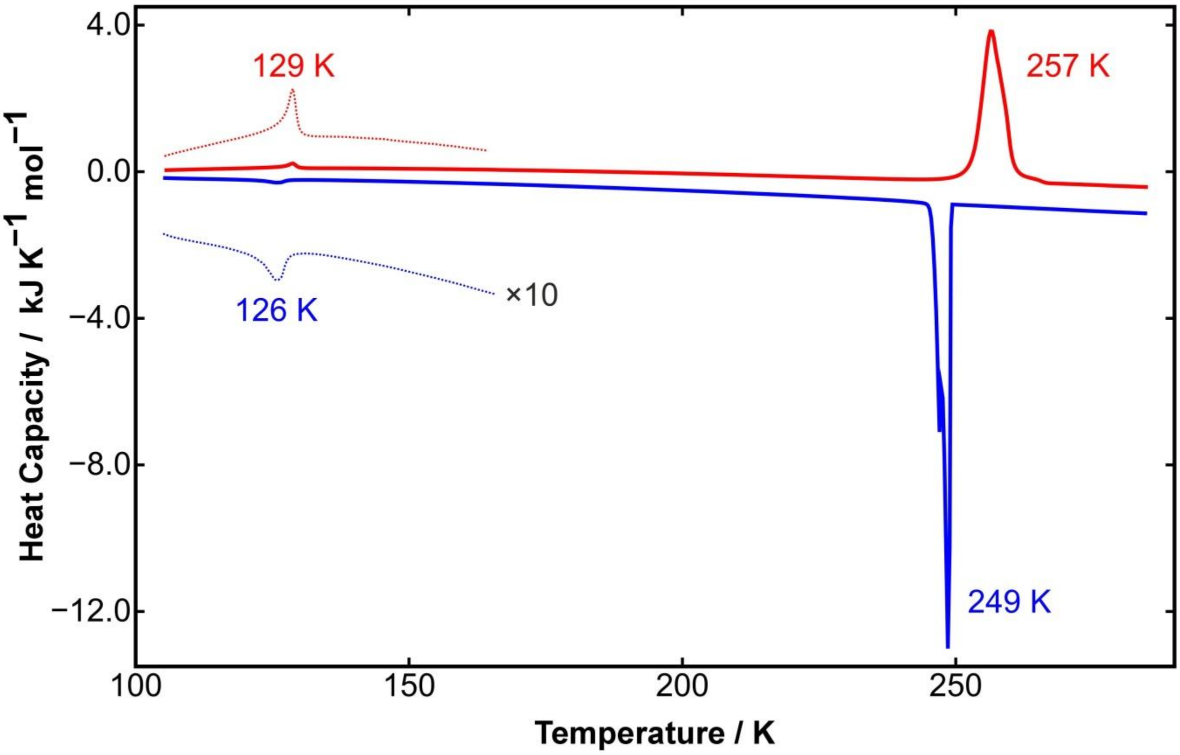

Figure S16 The curves show DSC data obtained on cooling (in blue) and heating (in red) for a sample of *p*-$C_6H_4Me_2$:C_6F_5H. A single solid-state phase transition is observed on cooling (126 K) and heating (129 K). The DSC curves for this phase transition are shown on an expanded (×10) scale given the intensity of the peaks due to the freezing and melting transitions at 249 K and 257 K, respectively.

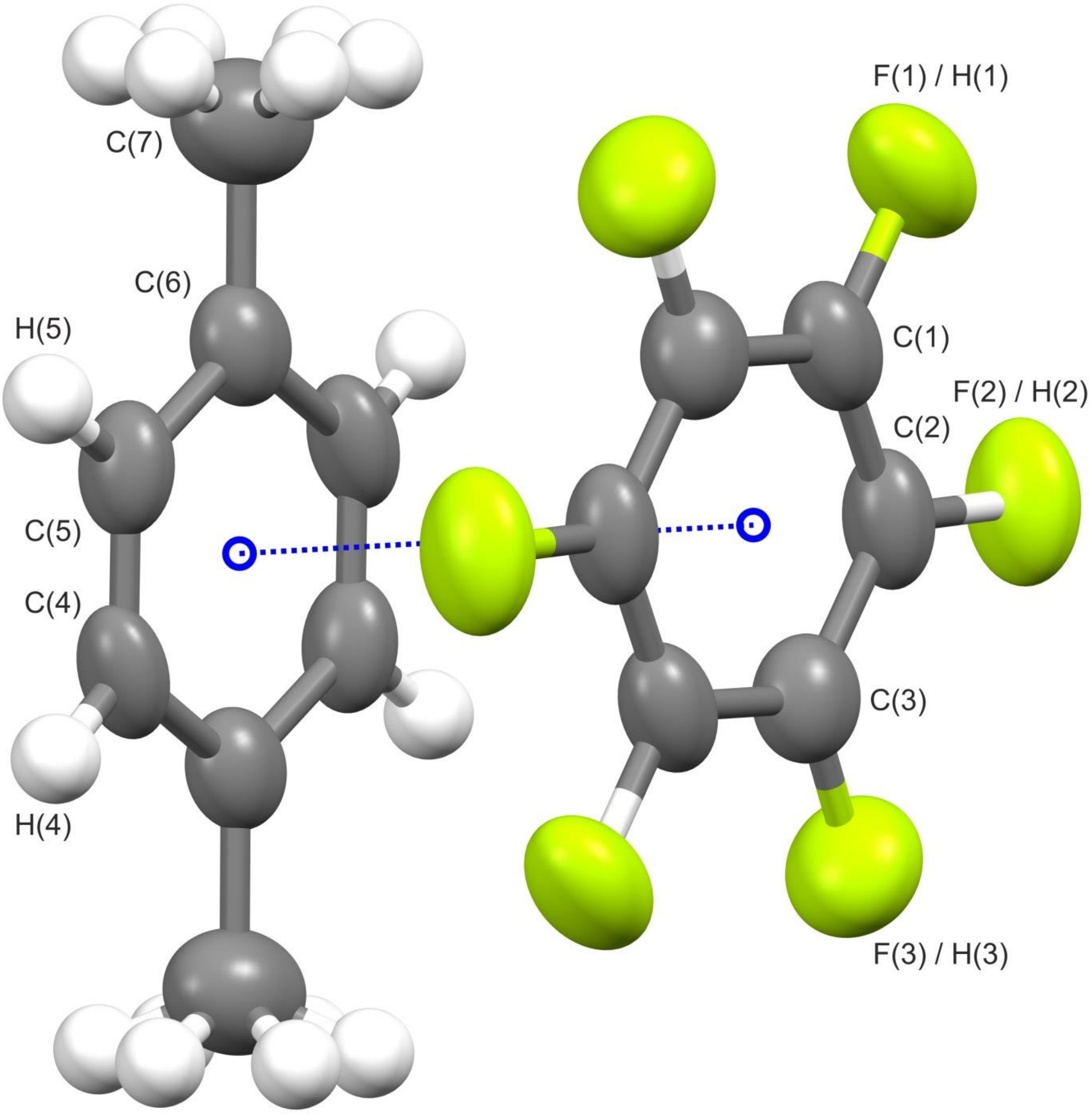

Figure S17 Asymmetric unit from the refined crystal structure of phase I of *p*-$C_6H_4Me_2$:C_6F_5H in space group $P\overline{1}$ at 160 K showing the crystallographic labelling of the atoms. H atoms are numbered according to the label of the atom to which they are bonded; disorder of the methyl groups is represented by the use of multiple H atoms which are not labelled. Unlabelled atoms in both molecules are symmetry related by inversion symmetry (shown as open blue circles) to the labelled atoms. The dotted blue line shows the column axis formed by the alternate stacking of *p*-$C_6H_4Me_2$ and C_6F_5H molecules.

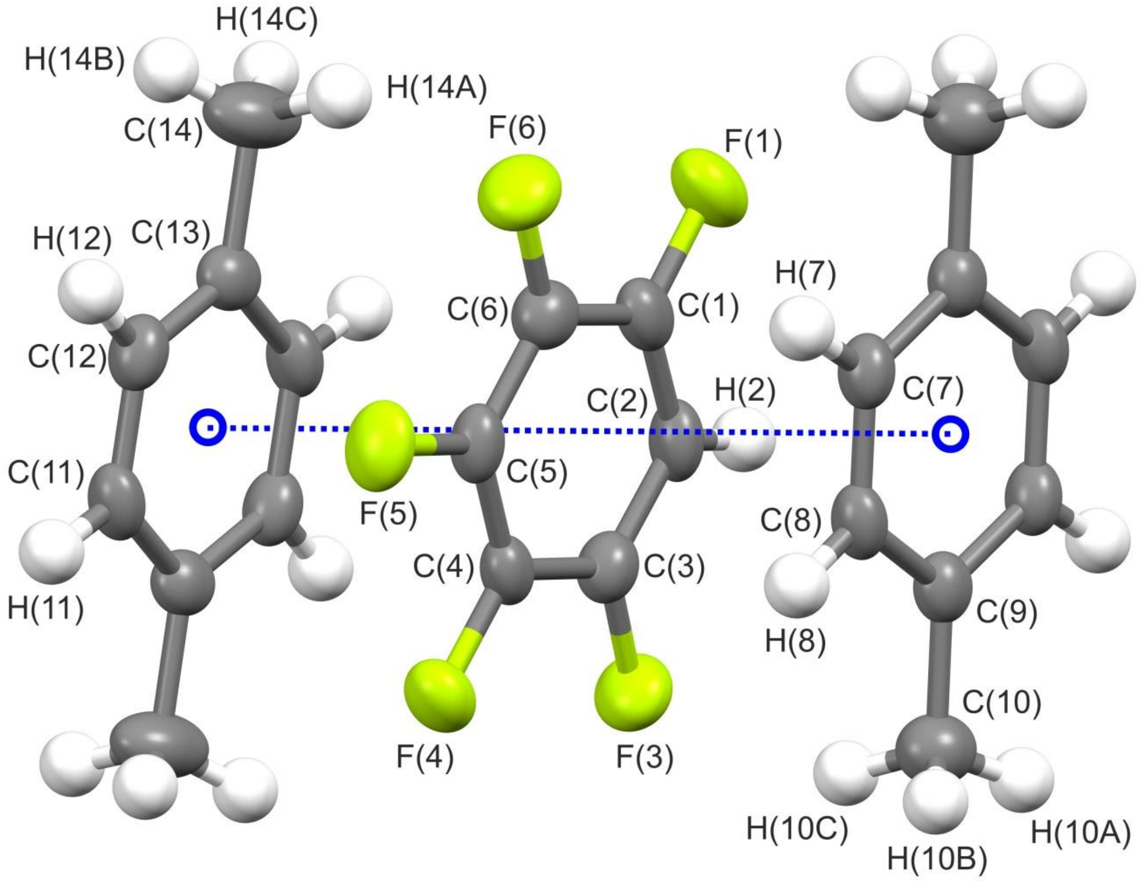

Figure S18 Asymmetric unit from the refined crystal structure of phase II of *p*-$C_6H_4Me_2$:C_6F_5H in space group $P\bar{1}$ at 120 K showing the crystallographic labelling of the atoms. H atoms are numbered according to the label of the atom to which they are bonded. Unlabelled atoms in the *p*-$C_6H_4Me_2$ molecules are symmetry related by inversion symmetry (shown as open blue circles) to the labelled atoms. In contrast to phase I, there is no crystallographic inversion symmetry at the centre of the C_6F_5H molecule. The dotted blue line shows the column axis formed by the alternate stacking of *p*-$C_6H_4Me_2$ and C_6F_5H molecules.
